# Generation and Characterization of Novel Pan‐Cancer Anti‐uPAR Fluorescent Nanobodies as Tools for Image‐Guided Surgery

**DOI:** 10.1002/advs.202400700

**Published:** 2024-06-06

**Authors:** Łukasz Mateusiak, Sam Floru, Timo W. M. De Groof, Janne Wouters, Noemi B. Declerck, Pieterjan Debie, Simone Janssen, Katty Zeven, Janik Puttemans, Cécile Vincke, Karine Breckpot, Nick Devoogdt, Sophie Hernot

**Affiliations:** ^1^ Laboratory for Molecular Imaging and Therapy Vrije Universiteit Brussel (VUB) MITH Laarbeeklaan 103 Brussels 1090 Belgium; ^2^ Faculty of Veterinary Medicine Small Animal Department Ghent University (UGent) Salisburylaan 133 Merelbeke 9820 Belgium; ^3^ Laboratory for Cellular and Molecular Immunology Vrije Universiteit Brussel (VUB) Pleinlaan 2 Brussels 1050 Belgium; ^4^ Myeloid Cell Immunology Lab VIB Center for Inflammation Research Pleinlaan 2 Brussels 1050 Belgium; ^5^ Laboratory for Molecular and Cellular Therapy Vrije Universiteit Brussel (VUB) Laarbeeklaan 103 Brussels 1090 Belgium

**Keywords:** fluorescence molecular imaging, fluorescence‐guided surgery, single‐domain antibodies, urokinase plasminogen activator receptor

## Abstract

Fluorescence molecular imaging plays a vital role in image‐guided surgery. In this context, the urokinase plasminogen activator receptor (uPAR) is an interesting biomarker enabling the detection and delineation of various tumor types due to its elevated expression on both tumor cells and the tumor microenvironment. In this study, anti‐uPAR Nanobodies (Nbs) are generated through llama immunization with human and murine uPAR protein. Extensive in vitro characterization and in vivo testing with radiolabeled variants are conducted to assess their pharmacokinetics and select lead compounds. Subsequently, the selected Nbs are converted into fluorescent agents, and their application for fluorescence‐guided surgery is evaluated in various subcutaneous and orthotopic tumor models. The study yields a panel of high‐affinity anti‐uPAR Nbs, showing specific binding across multiple types of cancer cells in vitro and in vivo. Lead fluorescently‐labeled compounds exhibit high tumor uptake with high contrast at 1 h after intravenous injection across all assessed uPAR‐expressing tumor models, outperforming a non‐targeting control Nb. Additionally, rapid and accurate tumor localization and demarcation are demonstrated in an orthotopic human glioma model. Utilizing these Nbs can potentially enhance the precision of surgical tumor resection and, consequently, improve survival rates in the clinic.

## Graphical abstract

This study details the generation and characterization of a panel of high‐affinity anti‐uPAR Nanobodies specific for various cancer types. The selected lead fluorescently‐labeled Nanobody demonstrates rapid and precise tumor localization and demarcation in an orthotopic human glioma model. These outcomes indicate the potential of these Nanobodies for applications in preclinical and clinical settings, providing real‐time guidance during surgical procedures.

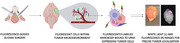

## 1 Introduction

Despite the considerable progress in cancer management over the last 30 years, surgery remains the basis of oncological treatment for localized tumors. Ideally, cancer surgery results in a complete resection of the tumor with achievement of negative resection margins, leading to a lower chance of tumor recurrence. To minimize the invasiveness and maximize the effectiveness of a surgical intervention, neo‐ and/or adjuvant therapies are often implemented to augment the treatment.^[^ ^1^ ^]^ To address the problem of reliable identification and localization of cancer cells in situ, several modalities have been developed to guide the surgeon during the procedure. The technology using electromagnetic radiation or ultrasonic waves to visualize and orient tumor location in reference to the anatomy of the patient is commonly referred to as image‐guided surgery (IGS).^[^ ^2^ ^]^

A lot of focus has recently been directed toward fluorescence imaging as a relatively inexpensive, highly sensitive, and easy to implement real‐time technology based on the detection of light emitted by an excited fluorophore in situ.^[^ ^3^ ^]^ Examples of clinically applied contrast agents are indocyanine green (ICG) and 5‐ALA‐derived metabolite protoporphyrin IX (PpIX), used respectively in liver tumor,^[^ ^4^, ^5^ ^]^ sentinel lymph node^[^ ^6^ ^]^ or tissue perfusion assessment,^[^ ^7^, ^8^ ^]^ and malignant glioma^[^ ^9^ ^]^ surgery. A shift toward more targeted agents has been observed in recent years with Cytalux (pafolacianine), a folate‐receptor‐directed near‐infrared (NIR) small molecule, being the first approved targeted tumor‐specific fluorescent contrast agent, used for visualization of ovarian^[^ ^10^ ^]^ and lung^[^ ^11^, ^12^ ^]^ cancer. Numerous ongoing clinical trials in this context are examining additional fluorescent molecular agents against receptors expressed by cancerous tissue.^[^ ^13^, ^14^, ^15^ ^]^ In the advanced stages of clinical trials, the tracers that are attracting the most attention include the following: bevacizumab‐IRDy800CW which targets VEGF (currently in phase III trials for invasive breast cancer, National Clinical Trial ID (NCT) 05939310); SGM‐101, which is the subject of investigation for CEA targeting (currently in phase III trials for colorectal cancer, NCT03659448); tozuleristide directed against cell‐surface chlorotoxin binding proteins (having completed phase 2/3 trials for pediatric CNS tumors, NCT03579602); and cathepsins‐targeting LUM015 (recently completing phase III trials for breast cancer, NCT03686215).

One of the current development strategies involves the generation of tracers against targets expressed on multiple cancer types, resulting in a broader tracer application, and making patient stratification unnecessary. For instance, the urokinase plasminogen activator receptor (uPAR), a cell‐membrane‐anchored biomarker characterized by elevated expression in several human tumors on both cancer and stromal components of the tumor microenvironment,^[^ ^16^, ^17^, ^18^, ^19^ ^]^ offers promising potential as a more general tumor target.^[^ ^20^, ^21^, ^22^ ^]^ uPAR's functional role has been correlated with tumor progression and angiogenesis, in particular through enhancing cellular proliferation, migration, invasion, and metastasis.^[^ ^16^, ^23^ ^]^ The imaging of uPAR tumor expression is pertinent to IGS due to its limited expression in physiological conditions in contrast to quickly remodeling, injured or inflamed tissues. In the context of IGS, the elevated expression of uPAR on the invasive edge of tumors renders it suited to clearly delineate and distinguish between healthy and diseased tissue.^[^ ^24^ ^]^

Different types of anti‐uPAR targeting moieties are being investigated for molecular imaging with 9‐mer AE105 peptide variants emerging as the frontrunners.^[^ ^25^, ^26^ ^]^ With their more intricate and robust structure, antibody‐derived fragments such as Nanobodies (Nbs)^[^ ^27^ ^]^ are being explored as an alternative platform to generate contrast agents for IGS owing to their favorable pharmacokinetic parameters. In the past, fluorescently‐ and/or radiolabeled Nbs have been reported as promising targeting moieties for molecular imaging, primarily in oncology.^[^ ^28^, ^29^, ^30^ ^]^ Unlike the larger (≈150 kDa) monoclonal antibodies, Nbs with their mid‐range molecular weight (12–15 kDa) and lack of the Fc region contribute to quick systemic clearance. This translates to a significantly shorter time interval between injection and highly‐specific imaging with sufficient tumor contrast. Additionally, their more robust structure offers high in vivo stability in contrast to short peptides (0.5–5 kDa).

In this study, we first generated and characterized in vitro anti‐uPAR Nbs targeting human (H), murine (M) and/or canine (C) homologues of the receptor. Next, we used radiolabeled Nb analogues to assess their biodistribution and show in vivo tumor targeting in subcutaneous tumor models in mice via nuclear imaging. Finally, we evaluated lead anti‐uPAR Nbs labeled with the NIR fluorophore s775z through fluorescence imaging in subcutaneous cancer models and demonstrated that a novel HuPAR‐targeting Nb enabled fast and highly specific molecular imaging of orthotopic human glioma.

## 2 Results

### 2.1 Generation and In Vitro Characterization of uPAR‐Specific Nbs

**Figure 1:**
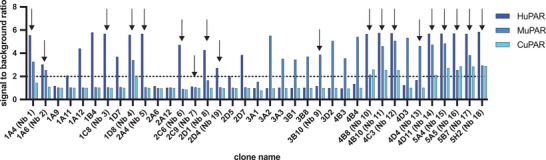
BE‐ELISA results for the 37 preselected clones, showing the ratio of measured signal between positive (H, M, and CuPAR protein) and control conditions (uncoated). Arrows indicate the nineteen clones selected for further in vitro evaluation.

**Figure 2:**
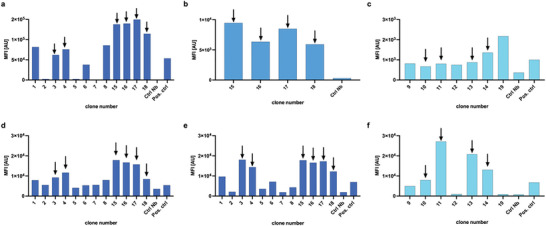
Mean fluorescent intensity (MFI) values obtained during flow cytometry screening of the selected Nbs on transduced HEK cells (a–c respectively H, C, MuPAR), and d) U87, e) HT29, and f) MC38 cells. Nbs selected for further in vivo evaluation are indicated with arrows.

Llama immunization yielded 75 unique anti‐uPAR Nb binders, from which 37 clones were picked based on the uniqueness of their sequences and their diverse representation across various families (the sequences of the family of the lead Nb 15 and Nb 13 – the only one of its family – are given in Figure S1, Supporting Information). Based on the results of the cross‐reactivity screening among the different uPAR homologues in a follow‐up BE‐ELISA (**Figure** **1**) and the estimation of their k_off_ ‐values via SPR (Table S1, Supporting Information), nineteen Nbs were selected for production in bacterial cultures. Further selection based on good production yields, high thermal stability, low nm‐affinities (K_D_<10 nm) and low k_d_‐values (Figure S2, Table S1, Supporting Information) as well as specific binding to both transduced HEK cells and naturally uPAR‐expressing cells (**Figure** **2**) resulted in choosing two binders for HuPAR, four for MuPAR, and four cross‐reactive with both H/CuPAR. These clones were advanced toward in vivo assessment.

### 2.2 Evaluation of In Vivo Biodistribution and Tumor Targeting of uPAR‐Specific Nbs via Nuclear Imaging

**Figure 3:**
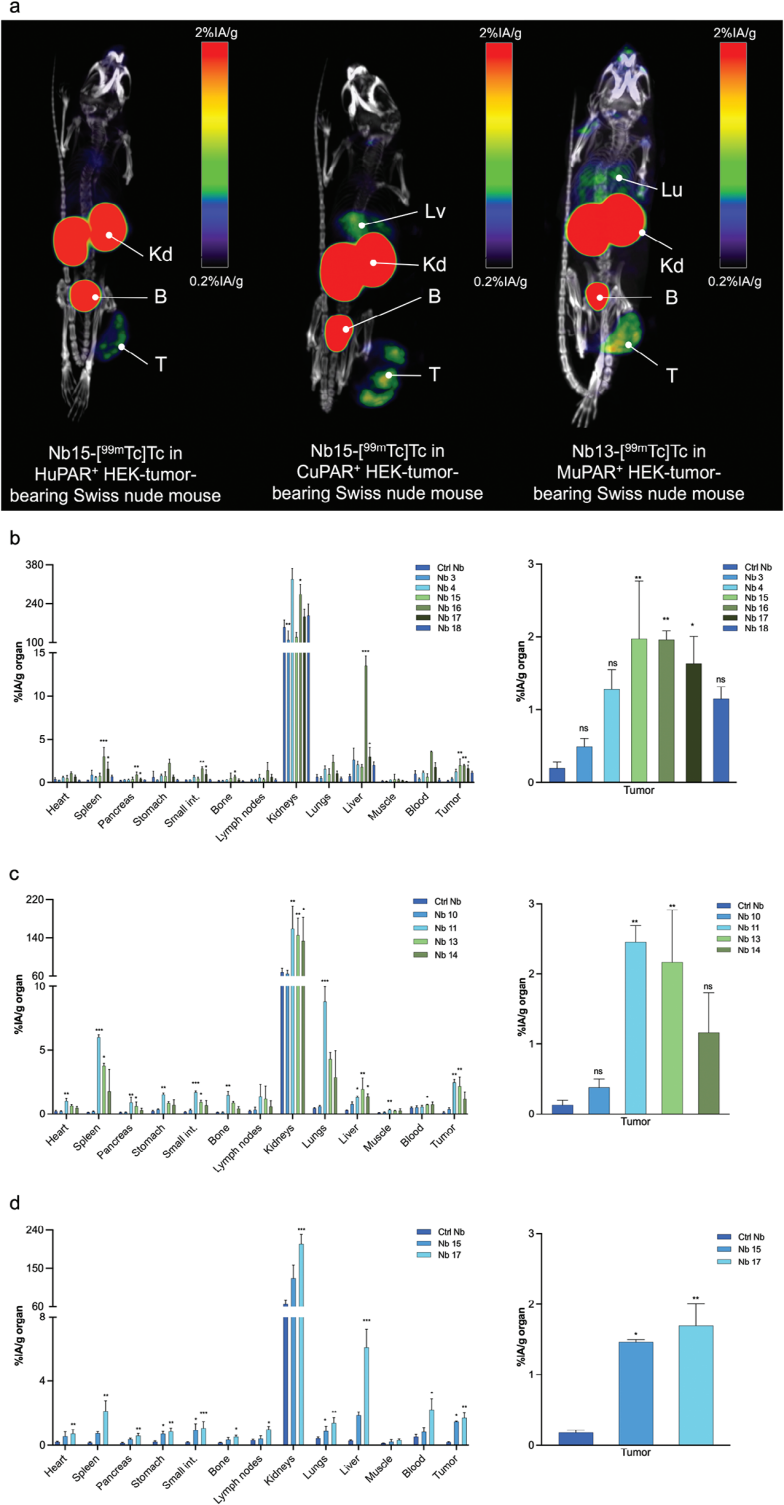
a) Biodistribution of ^99m^Tc‐labeled anti‐uPAR Nbs, in Swiss nude mice bearing subcutaneous uPAR‐transduced HEK tumors. Micro‐SPECT/CT images were obtained 1 h after intravenous injection of ^99m^Tc‐labeled Nbs. Arrows indicate T: tumor; B: bladder; Kd: kidneys; Lv: liver, and Lu: lungs. Ex vivo biodistribution and tumor targeting of the ^99m^Tc‐labeled anti‐uPAR Nbs and non‐targeting control Nb in Swiss nude mice subcutaneously bearing H, M or CuPAR transduced HEK tumors (b–d, respectively). Statistical analyses were performed using a Kruskal‐Wallis test followed by a Dunn's multiple comparisons test for the selection of the lead Nbs. Statistical significance was set at *p* < 0.05 (*p < 0.05, **p < 0.01, ***p < 0.001, ****p < 0.0001).

Based on SPECT/CT imaging and ex vivo biodistribution studies, most of the selected H and CuPAR‐binders displayed a typical biodistribution profile for Nbs (**Figure** **3**; and Table S2, Supporting Information), characterized by rapid blood clearance (≤ 1.16%IA g^−1^ at 1 h post‐injection), minimal uptake in normal organs and tissues, and significant kidney retention (ranging from 109 ± 33 to 328 ± 39%IA g^−1^). In contrast, Nb 16 and 17 exhibited relatively high non‐specific background signals, which prompted their exclusion from further investigation. Nb 15 showed significantly higher tumor uptake in HuPAR expressing HEK tumors (1.98±0.79%IA g^−1^) than the non‐targeting control Nb R3B23 (0.20 ± 0.08%IA/g), and similar uptake in  CuPAR expressing tumors (1.46 ± 0.03%IA g^−1^). Among MuPAR binders, Nbs 11 and 13 exhibited the highest tumor uptake as compared to R3B23 (respectively 2.46 ± 0.23 and 2.17 ± 0.74%IA g^−1^ vs 0.13±0.07%IA g^−1^, *p *< 0.05). Interestingly, the tumor uptake of the MuPAR binders correlated with the uptake in the lungs (8.83 ± 1.13 and 4.28 ± 0.54%IA g^−1^, respectively) and the spleen (6.02 ± 0.19 and 3.77 ± 0.19%IA g^−1^, respectively). This was attributed to the specific recognition of constitutively expressed uPAR in these organs (see below). The main focus on Nb 13 as a lead compound for anti‐MuPAR was driven by its minimal non‐specific uptake.

**Figure 4:**
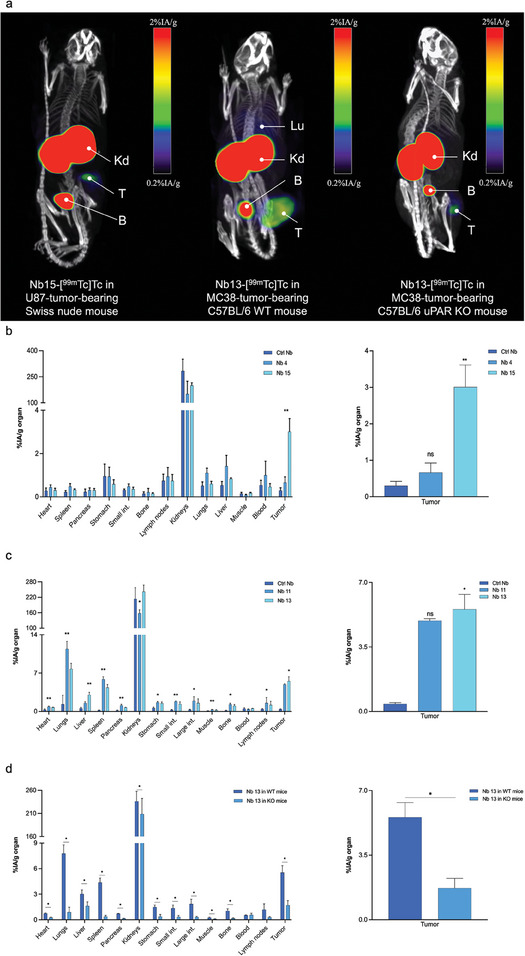
a) Biodistribution of ^99m^Tc‐labeled Nbs 15 or 13, in Swiss nude (left), WT (middle) or uPAR KO (right) C57BL/6 mice bearing subcutaneous U87 or MC38 tumors, respectively. Micro‐SPECT/CT images were obtained 1 h after intravenous injection of ^99m^Tc‐labeled Nbs. Arrows indicate Kd: kidneys; T: tumor; B: bladder; and Lu: lungs. Ex vivo biodistribution and tumor targeting of the b) anti‐HuPAR Nbs‐[^99m^Tc]Tc and c) anti‐MuPAR Nbs‐[^99m^Tc]Tc, together with radiolabeled non‐targeting control Nb. d) Comparison of Nb13‐[^99m^Tc]Tc biodistribution between WT and uPAR KO C57BL/6 mice bearing a syngeneic MC38 tumor. Statistical analyses were performed using a Kruskal‐Wallis test followed by a Dunn's multiple comparisons test for the selection of the lead Nbs. A Mann‐Whitney test was used for comparison of Nb13‐[^99m^Tc]Tc uptake between WT and uPAR KO mice. For all tests statistical significance was set at *p* < 0.05 (*p < 0.05, **p < 0.01, ***p < 0.001, ****p < 0.0001).

Next, the biodistribution and tumor targeting of the selected Nbs were evaluated in mice bearing subcutaneous U87 and MC38 tumors (**Figure** **4**; and Tables S3 and S4, Supporting Information), due to the high clinical relevance of surgical treatment of diffuse glioma and colorectal cancer. The biodistribution of the HuPAR binders closely mirrored previous findings, although tumor uptake of Nb 15 was higher and reached the value of 3.01 ± 0.60%IA g^−1^ ex vivo (Figure 4b; and Table S3, Supporting Information). For the MuPAR binders, tumor uptake was also considerably higher for both tested Nbs as compared to the uptake in MuPAR‐transduced HEK tumors, with values up to 5.55 ± 0.80%IA g^−1^ for Nb 13 (Figure 4c; and Table S4, Supporting Information). This was attributed to the possibly lower uPAR expression on the transduced HEK293T cells than on the MC38 cells (Figure 2c,f). Furthermore, it is hypothesized that for the Nbs recognizing the murine homologue of uPAR, there is a significant contribution of the uPAR expressed on the stromal cells in the tumor microenvironment, on top of the uPAR expressed by the cancer cells themselves. To confirm this hypothesis, the tumor uptake of Nb 13 was investigated in uPAR gene knock‐out (KO) C57BL/6 mice bearing a syngeneic subcutaneous MC38 tumor. As hypothesized, in this model, MC38 tumor uptake significantly decreased as compared to the uptake in WT C57BL/6 mice (5.55 ± 0.80 vs 1.71 ± 0.53%IA g^−1^ ex vivo, *p *< 0.05, Figure 4a,d; and Table S4, Supporting Information). Moreover, signal in the lungs and spleen was also reduced to background levels, confirming that the uptake in these organs of WT mice is indeed uPAR‐specific. Based on all in vivo results, in combination with the previously obtained in vitro data, Nb 15 and Nb 13 were selected as final lead compounds for respectively HuPAR/CuPAR and MuPAR targeting.

### 2.3 In Vivo Evaluation of Fluorescently‐Labeled uPAR‐Specific Nbs

**Figure 5:**
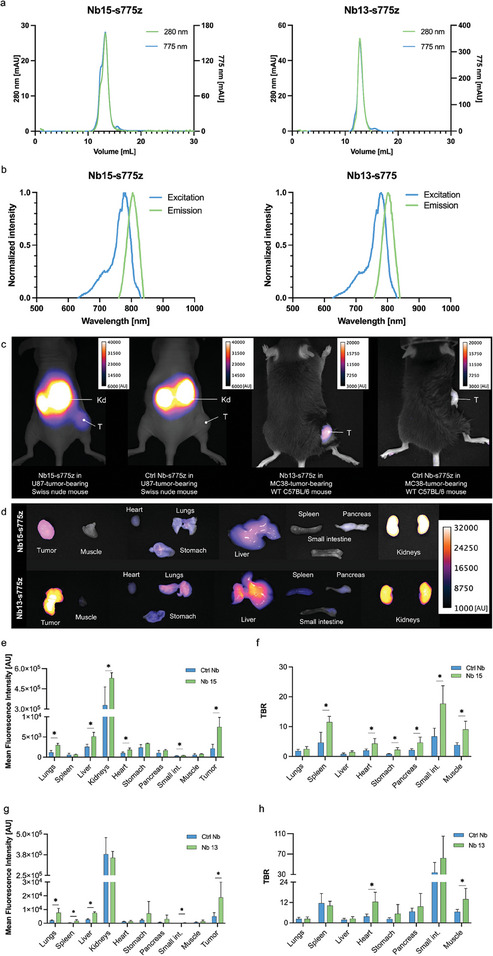
a) Chromatograms and b) excitation and emission spectra of Nb15‐ and Nb13‐s775z, respectively, representing desired profiles for randomly labeled Nb. c) In vivo fluorescence imaging of Nb15‐ (left) and Nb13‐s775z (right) with corresponding non‐targeting (control) Nb R3B23‐s775z in mice bearing a subcutaneous U87 or MC38 tumor, respectively. Fluorescence images were obtained 1 h after intravenous injection of labeled Nbs. Arrows indicate Kd: kidneys and T: tumor. d) Ex vivo fluorescence imaging of organ biodistribution of s775z‐labeled Nbs 15 or 13. MFIs and TBRs of dissected organs of Nb15‐s775z e,f) and Nb13‐s775z g,h) grouped together with values obtained from corresponding controls. Statistical analyses were performed using a Mann‐Whitney test for comparison of MFI and TBR values between targeting and control Nb. Statistical significance was set at *p* < 0.05 (*p < 0.05).

Both lead compounds were subsequently labeled with the NIR fluorescent dye s775z via amine‐reactive conjugation chemistry. A degree of labeling of 1 on average was obtained and spectral characteristics of the dye remained close to those of the free dye (ex/em maxima: 775/804 and 780/802 nm for Nb 15 and Nb 13, respectively (**Figure** **5**a,b). Following tracer injection into respectively U87 and MC38‐tumor‐bearing mice, in vivo images at 1 h post‐injection and ex vivo images of dissected organs showed clear uptake of the tracers in the tumor with low background, except for the kidneys (renal clearance) (Figure 5c,d). Ex vivo TBRs (calculated for different organs) reached high enough values to be relevant for IGS for both Nbs 15 and 13, and were considerably higher than for the control Nb (Figure 5f,h).

### 2.4 Proof‐of‐Concept of Intraoperative Tumor Imaging with Fluorescently‐Labeled Anti‐uPAR Nb

**Figure 6:**
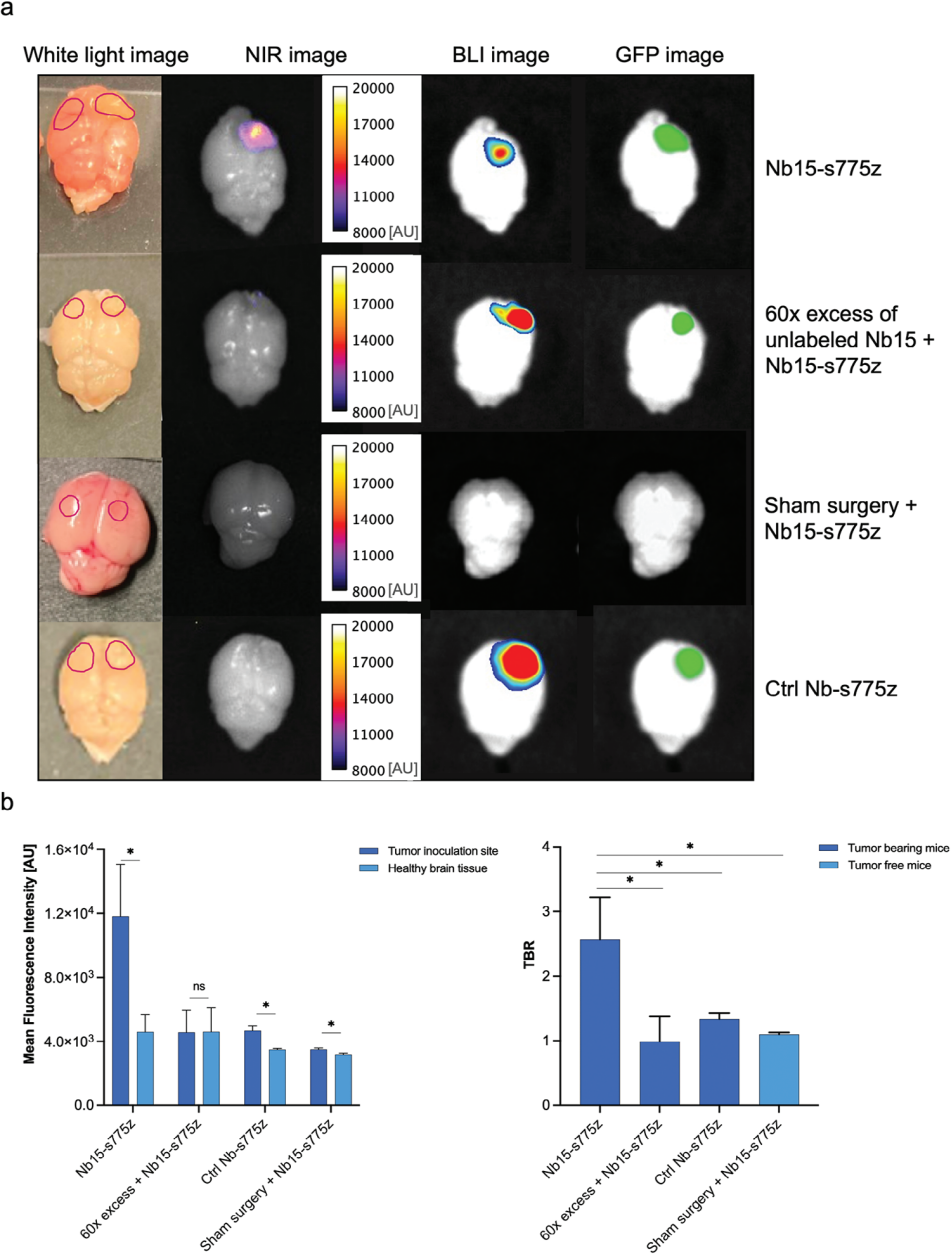
a) Ex vivo brain imaging 1 h after intravenous injection of fluorescently‐labeled Nbs in U87 orthotopic tumor‐bearing or sham‐operated mice. Regions of interest (ROIs) were drawn based on the GFP image (or injection site for sham surgery) and are indicated on white light images (injection sites and healthy tissues are indicated on the right and left hemispheres, respectively). b) MFIs of tumors and healthy brain tissue and quantified TBRs. Statistical analyses were performed using a Mann‐Whitney test. Healthy brain tissue was used as a control for MFI. TBR values were compared between targeting Nb and control conditions. Statistical significance was set at *p* < 0.05 (*p < 0.05).

As proof‐of‐concept for the use of Nb 15 as fluorescent tracer for image‐guided surgery, intraoperative imaging of brain cancer was mimicked in an orthotopic GFP^+^/FLuc^+^ U87 tumor model. After removal of the skull, NIR fluorescent signals could clearly be observed in tumor‐bearing mice injected with Nb15‐s775z using an intraoperative fluorescence imaging system. Contrarily, no signal was detected in mice injected with the control Nb, mice injected with an excess of unlabeled Nb 15, or sham‐operated mice. The fluorescent signal of Nb15‐s775z colocalized with the BLI and GFP signals (**Figure** **6**a). Ex vivo TBRs, calculated using healthy brain tissue as background, reached 2.57 ± 0.65 for Nb15‐s775z, and 0.99 ± 0.39 or 1.34 ± 0.09 for mice injected with excess of unlabeled Nb 15, or s775z‐labeled control Nb, respectively. TBR for sham‐operated mice was determined at 1.10 ± 0.03. All the TBRs calculated for the control groups were significantly lower than the TBR of Nb15‐s775z (Figure 6b).

## 3 Discussion

In this study, we aimed to develop and validate a fluorescent Nb conjugate to enhance the visualization of tumors during IGS through their uPAR expression. Ultimately, we aim to improve the completeness of tumor resection leading to a reduced tumor recurrence, and consequently higher patient survival rate and better quality of life.

Nb 15, an anti‐human uPAR targeting Nb, was selected as lead compound for potential clinical translation as it showed high tumor uptake and minimal non‐specific targeting in various tumor models, as evidenced by both in vivo SPECT/CT and fluorescent imaging data. The variation in tumor uptake can be attributed to differing levels of uPAR expression between transduced and naturally expressing tumor cells. However, because Nb 15 does not cross‐react with the murine homologue of uPAR, it is not possible to evaluate the so‐called “on‐target, off‐tumor uptake” in organs that constitutively express uPAR in mouse models. To address this limitation, Nb 13, which targets MuPAR, was also selected to serve as a surrogate for Nb 15 during preclinical studies. Nb 13 not only exhibited specific tumor targeting, but also displayed specific uptake in the lungs and spleen. The physiologically elevated uPAR expression in these organs, which has been previously documented in the literature,^[^ ^31^, ^32^ ^]^ may limit the surgical application of uPAR‐targeting Nbs (and by extension also other uPAR‐targeting contrast agents), in particular for lung cancer. Additionally, the significantly lower tumor uptake we observed in uPAR KO mice compared to WT mice supports earlier findings that tumor stroma highly contributes to the overall expression of uPAR in the tumor and consequently to the tracer uptake.^[^ ^16^, ^17^, ^18^, ^19^ ^]^ Hence, we anticipate even higher tumor uptake of the lead anti‐HuPAR Nb 15 in humans compared to the proposed mouse models. The ability of Nb 15 to cross‐react with the canine equivalent of uPAR opens the possibility of evaluating this aspect using a more relevant animal model with spontaneous tumors.^[^ ^33^ ^]^ This intermediate step offers a more realistic model for the human scenario, before making more substantial investments for clinical translation. Furthermore, it creates the potential for utilizing this Nb also for veterinary applications.

The assessment of biodistribution and tumor targeting via nuclear imaging, prior to the evaluation of fluorescently‐labeled Nbs, has enabled us to better anticipate in vivo behavior and obtain more quantitative data on organ uptake compared to relying solely on fluorescent imaging. It would nonetheless also be interesting to further explore the potential application of a uPAR‐targeting Nb in nuclear medicine, mirroring the use of the radiolabeled anti‐uPAR peptide AE105, which exhibits nanomolar affinity, comparable to Nb 15. Previously, following the safety assessment of ^64^Cu‐DOTA‐AE105 for PET imaging,^[^ ^34^ ^]^^68^Ga‐NOTA‐AE105 has been successfully used for risk stratification and evaluation of cancer aggressiveness in patients with neuroendocrine neoplasms and prostate cancer.^[^ ^35^, ^36^ ^]^ It has also been suggested as a valuable adjunct to ^18^F‐FDG PET imaging, offering supplementary non‐invasive tumor characterization, following a trial in patients with HNSCC.^[^ ^37^ ^]^ We acknowledge that to enhance sensitivity of the generated Nbs, transitioning to a PET imaging tracer labeled with isotopes like ^68^Ga or ^18^F would be necessary, rather than using ^99m^Tc.^[^ ^38^, ^39^ ^]^

Given the well‐known negative effect that fluorescent dyes can have on the tracer's biodistribution,^[^ ^40^, ^41^, ^42^ ^]^ we opted for the novel NIR cyanine‐based fluorophore s775z. The choice of s775z was guided by the improved biodistribution we observed in comparison to the previously used NIR dye IRDye800CW, in particular in case of a random conjugation strategy.^[^ ^40^ ^]^ We recently demonstrated that selecting a self‐shielded and charge‐balanced dye minimized undesirable dye‐driven interactions in vivo and enabled imaging with Nbs as soon as 1 h post‐injection, while keeping liver uptake to a minimum.^[^ ^43^ ^]^ Additionally, s775z proved to be less prone to photobleaching compared to other NIR dyes.^[^ ^44^ ^]^ With the exception of a slightly more elevated signal in the liver for the fluorescently‐labeled Nbs, the ex vivo biodistribution data of fluorescent Nbs closely mirror the results obtained with the radiolabeled Nbs.

In a final in vivo proof‐of‐concept experiment, we demonstrated that the lead anti‐HuPAR Nb 15 efficiently visualized and clearly delineated orthotopic human glioma tumors within 1 h post‐injection, with a mean in situ TBR value of ≈2.6. Importantly, neither the non‐targeting control Nb nor Nb15‐s775z after injection of an excess of unlabeled Nb 15 showed any relevant signal. These results confirm the specificity of the chosen tracer. Additionally, the lack of statistically significant uptake of Nb15‐s775z in sham‐operated mice indicates that neither the surgical procedure nor the possible post‐inoculation wound‐healing‐related uPAR expression contributed to the tracer uptake in brain tumors. The predominant signals originating from the kidneys and urinary bladder represent the biggest limitations of using fluorescent‐Nb‐based tracers. Nevertheless, it is essential to consider that the distances between potential organs of interest, such as the brain, and the urinary system in humans are significantly greater than in mice, and that the thicker renal fascia will largely attenuate the renal fluorescent signal. We would however like to acknowledge that a potential limitation of our in vivo studies is the relatively small sample size, with only four animals included per tested group.

While 5‐ALA has gained clinical approval for guiding brain surgery,^[^ ^9^, ^45^, ^46^, ^47^ ^]^ it still faces challenges in fluorescence imaging, including uneven signal distribution that correlates with the tumor's grade, restricted penetration depth, and vulnerability to photobleaching. As alternative to 5‐ALA, a recent clinical study investigated a novel peptide‐based agent as the first NIR uPAR‐targeted contrast agent for visualization of brain cancer. Injected ICG‐conjugated peptide (AE105) allowed clear delineation of the tumor, however, the tracer needed to be injected 6 h prior to surgery for optimal results.^[^ ^48^ ^]^ Nevertheless, the limitation of this study was reporting results for a single patient. Follow‐up studies, which involved additional patients, have been conducted but the elaborate results are yet to be published [148]. A parallel preclinical study of a more hydrophilic alternative of this peptide (AE344) labeled with IRDye800CW, allowed orthotopic glioma resection in mice after 3 h following the intravenous injection.^[^ ^49^ ^]^ The authors also reported a more complete tumor resection than with 5‐ALA. While the reported TBR with their tracer is higher than for our Nb 15 (6.6 vs 2.6), they still observe TBR values significantly exceeding background levels after blocking or using an inactive probe, which might indicate limited specificity. In non‐brain‐related orthotopic models, Baart et al. compared the application of a highly specific high‐affinity anti‐uPAR antibody (MNPR‐101) and its Fab and F(ab’)_2_ fragments, respectively at 96, 48, and 36 h in head and neck, pancreatic and colorectal cancers. Using smaller antibody fragments allowed visualization of the primary tumors at earlier time‐points without statistically significant difference between the TBRs (ranging from 2.3 ± 1.0 and 3.3 ± 1.2 in pancreatic cancer to 5.4 ± 0.8 and 4.9 ± 1.1 in colorectal cancer for Fab and F(ab’)_2_, respectively). However, the peak fluorescence intensity was reported to be lower for the smaller targeting moieties.^[^ ^50^ ^]^ Previously, the same group has also shown potential to highlight cervical lymph node metastases in the HNSCC orthotopic model with the highest TBRs (4.9 ± 0.6 compared to 1.6 ± 0.1 for control at 72 h) obtained between 72 and 96 h when using a hybrid version of MNPR‐101 labeled with NIR dye ZW‐800‐1 and ^111^In.^[^ ^51^ ^]^ Nevertheless, drawing conclusions for different tracers and different tumor models necessitates direct comparison and should take differences in photophysical properties of the fluorophores and imaging systems into consideration.

The positive outcome of this proof of principle study indicates that Nb15‐s775z is the first anti‐uPAR fluorescent tracer granting highlighting of cancer cells as soon as 1 h post‐injection. This short time interval is an important advantage for IGS, as the time between tracer injection and beginning of surgery is preferentially kept as short as possible. What is more, since uPAR is expressed in multiple types of cancer, the generated compound offers potential to be utilized for the targeting of not only brain tumors, but also adenocarcinomas of the pancreas, colorectum, stomach, cervix, as well as carcinomas of esophagus, head and neck, and breast among many others.^[^ ^16^, ^52^ ^]^ We acknowledge that the tracer we developed requires additional assessment in other cancer types to verify this statement.

One of the limitations of uPAR as a receptor targeted during the surgery is possible on‐target off‐tumor uptake because of the locally invasive character of surgical intervention as its expression increases during tissue remodeling and wound healing, especially in the case of subsequent surgeries. Thereby, uPAR is generally recognized to be process‐specific rather than restricted to specific cell lines.^[^ ^25^ ^]^ Consequently, it is anticipated that under different conditions, such as varying tumor sizes or degrees of invasiveness, the expression levels of uPAR may fluctuate. Additionally, a relatively low number of copies^[^ ^53^ ^]^ and partial or full cleavage of this membrane‐associated protein^[^ ^16^, ^54^ ^]^ could be possible hurdles for low‐affinity moieties or tracers targeting the shed domains specifically. However, none of these concerns proved to be application‐restrictive in case of the studies we designed and discussed in this paper. Based on our experience and previously reported results, we speculate that uPAR holds remarkable potential for IGS‐related applications.

## 4 Conclusion

We report here the development and preclinical validation of Nbs that can specifically visualize uPAR expression through in vivo nuclear and fluorescence imaging techniques. In particular, the lead compound Nb 15, recognizing HuPAR, is relevant toward future clinical translation, e.g., as contrast agent in nuclear imaging or to guide surgical procedures in real‐time. In addition, the lead MuPAR‐specific Nb 13 provided us with better insights on the pharmacokinetic profile that can be expected for human‐targeting uPAR Nbs (e.g., Nb 15) in patients, while this cannot be investigated in mice because of the lack of cross‐reactivity. Hence low, but non‐negligible on‐target, off‐tumor signal can be expected in spleen and lungs. Moreover, as uPAR‐expression originating from the stromal compartment of the tumor contributes considerably to the total detected signal in the cancer lesions, the tumor signal measured for HuPAR‐targeting Nbs in a mouse model is most likely an underestimation as compared to the signal that can be expected in patients.

## 5 Experimental Section

### Anti‐uPAR Nb Generation, Production, and In Vitro Screening

A llama was immunized with both H and M recombinant uPAR extracellular domain protein according to a standardized protocol,^[^ ^55^, ^56^, ^57^, ^58^ ^]^ and B‐lymphocytes were collected. Gene sequences encoding for the variable domains of the heavy‐chain only Abs were isolated and amplified to create a library of affinity‐matured Nbs. Subsequently, four rounds of biopanning were performed on immobilized H, M, or CuPAR, and bacterial extracts (BEs) were prepared for surface plasmon resonance (SPR) off‐rate screenings (k_off_) and ELISA testing as described previously.^[^ ^55^ ^]^ The DNA sequences of a first panel of selected anti‐uPAR Nbs were recloned into an expression vector and Nbs were produced in 1 L *E. coli* WK6 cultures. The Nbs were purified from the periplasm via osmotic shock treatment, immobilized metal affinity chromatography, and size‐exclusion chromatography. The non‐targeting control Nb, R3B23, was produced in the same manner.^[^ ^55^ ^]^ The thermal stability of all produced Nbs was determined using a thermal shift assay. Kinetic binding parameters (k_a_, k_d_, K_D_) toward recombinant H, M, or CuPAR antigens were determined via SPR and specific binding to cell surface expressed uPAR was evaluated via flow cytometry on either uPAR‐transduced HEK cells or mouse and human cancer cell lines naturally expressing uPAR (MC38, HT29, U87).^[^ ^59^ ^]^

### In Vivo Biodistribution of Technetium‐99m‐Labeled Nbs

After in vitro characterization, a second selection of Nbs was labeled with [^99m^Tc]Tc on their carboxy‐terminal His6 tag via tricarbonyl chemistry^[^ ^60^ ^]^ and intravenously injected in the mice (*n* = 4 per group) for in vivo biodistribution studies. Different cell lines (H, M, or CuPAR transduced HEK293T cells, MC38 or U87 cells) were subcutaneously inoculated into mice (Swiss nude Crl:NU(Ico)‐Foxn1^nu^, C57BL/6 WT, or C57BL/6 uPAR KO mice^[^ ^61^, ^62^ ^]^) and allowed to grow till 150–250 mm^3^. 30–60 MBq of ^99m^Tc‐labeled Nb was administered intravenously, and 1 h later, the mice underwent imaging using microSPECT/CT (VECTOR^+^ system, MILabs). After the SPECT/CT scan, mice were killed and the radioactivity in each collected organ was measured by gamma counting and expressed as percentage injected activity per gram of tissue (%IA/g). The biodistribution of each Nb was compared to that of the control Nb. All animal experiments were conducted in the accredited laboratory (license number LA1230272) under approval of the ethical commission for animal experimentation (ECD) of the Vrije Universiteit Brussel (projects 19‐272‐10, 20‐272‐13, 20‐272‐14, 21‐272‐06, 20‐394‐1).

### In Vivo Evaluation of Fluorescent Anti‐uPAR Tracers

The selected lead Nb binder for each species (Nb 15 and Nb 13 for H/C and MuPAR, respectively) was labeled randomly with the fluorescent dye s775z on primary amines using N‐hydroxysuccinimide ester reaction chemistry.^[^ ^40^ ^]^ Purity, concentration, degree of labeling, and spectral characteristics were assessed prior to further in vivo studies. To compare the biodistribution and tumor uptake of s775z‐labeled uPAR Nbs with that of the control Nb, 2 nmol of Nb was intravenously injected in mice bearing subcutaneous MC38 and U87 tumors. One hour post‐injection, mice were subjected to imaging using the NIR fluorescence camera Fluobeam (Fluoptics) in a dark setting. Immediately after in vivo imaging, mice were killed, and organs of interest were imaged ex vivo.

Finally, the anti‐HuPAR Nb15‐s775z was tested in an orthotopic brain tumor model (U87 FLuc^+^/GFP^+/^). Its performance was compared to i) Nb15‐s775z injected 30 min after preinjection of 60 times molar excess of unlabeled Nb 15, ii) control Nb R3B23‐s775z, and iii) sham‐operated mice injected with Nb15‐s775z. One hour after injection of the fluorescent tracer, mice were killed, and their brains were imaged ex vivo using Fluobeam. Additionally, ex vivo bioluminescence and GFP imaging were performed using the PhotoIMAGER Optima (Biospacelab).

Images were analyzed using ImageJ software (NIH, Bethesda, MD, USA).^[^ ^63^ ^]^ Regions of interest (ROIs) were determined based on the full organ or tumor shape in white light after organ resection. For brain imaging, the GFP signal served as the tumor shape reference, with similarly sized ROIs placed on the opposite side of the organ to determine the background signal. Mean fluorescence intensity (MFI) was measured and tumor‐to‐background ratios (TBR) were calculated by dividing the tumor signal by the background organ signal.

### Statistical Analyses

Statistical analyses were performed using Prism software v.9.4.1. The Kruskal‐Wallis test with Dunn's multiple comparisons test was used to analyze radioactive Nb uptake in tumor and organs compared to control Nb. One‐tailed Mann‐Whitney test was used for tumor uptake comparisons in uPAR KO versus WT mice and fluorescently‐labeled Nbs (MFI and TBR) versus control R3B23‐s775z. The results were described as mean ± SD. Each animal experiment included *n* = 4 mice per group. Significance was set at p<0.05 (*p < 0.05, **p < 0.01, ***p < 0.001, ****p < 0.0001).

### Ethics Approval

This study was performed in line with the directive 2010/63/EU transposed into Belgian law in a new Royal Decree in 2013. Approval was granted by the Ethics Committee on Animal Experimentation of the Vrije Universiteit Brussel (VUB) (projects 19‐272‐10, 20‐272‐13, 20‐272‐14, and 21‐272‐06). As required by Belgian law, the VUB's Animal Ethics Committee independently evaluates research projects submitted to it by researchers. It can approve or reject projects and must also critically‐evaluate these projects after completion. It also draws up ethical criteria for animal experiments and advises researchers and staff on them.

## Conflict of Interest

With the exception of Mr. Lukasz Mateusiak, Dr. Timo De Groof, Prof. Nick Devoogdt and Prof. Sophie Hernot, none of the authors have conflicts of interest or financial disclosures. The aforementioned authors specifically acknowledge that they hold a patent for Anti‐urokinase plasminogen activator receptor immunoglobulin single variable domains, under EP22199885.9.

## Supporting information

Supporting Information

## Data Availability

The data that support the findings of this study are available from the corresponding author upon reasonable request.
