# Supplementary material for: Generation and Characterization of Novel Pan‐Cancer Anti‐uPAR Fluorescent Nanobodies as Tools for Image‐Guided Surgery
Source: Adv Sci (Weinh). 2024 Jun 6;11(30):2400700. doi: 10.1002/advs.202400700 (PMC11321701; doi:10.1002/advs.202400700)
Supplement: Supplementary file 1 — Supporting Information [file ADVS-11-2400700-s001.docx]

# Supporting Information

# Supporting Experimental Section

# Anti-uPAR Nb generation

All Nbs were generated and produced according to standard operating procedures,^[54–57]^ unless stated otherwise. Briefly, a llama was immunized with recombinant H or MuPAR antigen (Sino Biological, Beijing, China) according to a standardized vaccination scheme. After 6 weeks, B-lymphocytes were collected from peripheral blood and the mRNA of the heavy-chain only Ab (HCAb) was extracted to generate cDNA using reverse transcriptase. Subsequently, the gene sequence encoding for the antigen binding fragment of the HCAbs was amplified by PCR. Next, the Nb cDNA was ligated into a pMECS-GG vector (introducing a carboxyterminal hemagglutinin and hexahistidine (His6) tag) in fusion with the phage gene III and transformed into E. coli cells to generate a library (library 161) according to the Golden Gate technology, which were then phage displayed upon infection with M13 VCS helper phages.^[63]^ Biopannings were performed in parallel on immobilized H, M or C recombinant uPAR (CuPAR was produced by U-Protein Express BV, Utrecht, The Netherlands) in four consecutive rounds.^[54]^ 0.50 μg (for the first two rounds of biopanning) or 0.25 μg (for the final two rounds of biopanning) in PBS (pH 7.4, Gibco, Thermo Fisher Scientific, Waltham, MA, USA) of antigen was coated in 96-well MaxiSorp plate (Thermo Fisher Scientific) overnight at 4°C. After rounds 2-4, individual colonies were picked randomly to prepare bacterial extracts (BEs). Colonies were grown in 96-deep-well plates (Thermo Fischer Scientific) in 1 mL 2X YT medium supplemented with D-glucose and ampicillin sodium (final concentrations 0.1% and 100 μg/mL, respectively). At OD_600_ = 0.6, expression was induced with 1 mM isopropyl β-D-1-thiogalactopyranoside (IPTG). After 4 h incubation at 37°C, plates were spun down, medium removed, and cells were lysed via overnight storage at -80°C. After resuspension in 100 µl PBS and centrifugation to remove cell debris, these extracts were used in ELISA and for surface plasmon resonance (SPR) off-rate screenings (k_off_).

# ELISA

96-well MaxiSorp plates were coated with 0.20 μg of H, M or CuPAR in PBS (pH 7.4) overnight at 4°C. The next day, wells were washed 5 times with PBST buffer (PBS + 0.05% Tween 20), blocked with 2% skimmed milk in PBS suspension for 1 h and washed again with PBST. Subsequently, a solution of 20 µL of the tested BEs in 80 µL of PBS was added to coated and uncoated (negative control) wells and incubated for 1 h at room temperature. Nbs were detected with mouse anti-hemagglutinin-mAb (1:2000; clone 16B12, BioLegend, San Diego, CA, USA) and alkaline-phosphatase-conjugated anti-mouse-IgG mAb (1:2000; A90-116AP, Bethyl Laboratories, Montgomery, TX, USA). All antibodies were diluted in PBS and allowed to bind for 1 h at room temperature. Between each incubation step, wells were washed 10 times with PBST. For chemiluminescent detection, p-nitrophenyl phosphate (PNPP) substrate solution (2 mg/mL in AP buffer; 100 mM NaCl, 100 mM Trizma base, 50 mM MgCl_2_ **×** 6H_2_O) was added and the signal was measured at OD_405_ using the VersaMax ELISA Microplate Reader (Molecular Devices, Downingtown, PA, USA) at 2, 10, 30 and 60 min timepoints. The binding was determined based on the signal ratio of the positive and negative wells.

# Anti-uPAR Nb production

Anti-uPAR Nb DNA fragments were cloned into a pHEN6 expression vector to introduce a carboxyterminal His6 tag and Nbs were produced in 1 L E. coli WK6 cultures in Terrific Broth medium supplemented with ampicillin sodium, D-glucose and MgCl_2_ (final concentrations 100 μg/mL, 0.1% and 2 mM, respectively). Nb expression was induced at OD_600_ = 0.6 with 1 mM IPTG overnight at 28°C. Periplasmic extracts (PEs) containing the soluble Nbs were collected from E. coli after osmotic shock treatment. Briefly, following the overnight Nb expression in WK6 E. coli, bacterial cell pellets were harvested and resuspended in 12 mL TES buffer (0.2 M Tris pH 8.0, 0.5 mM EDTA, 0.5 M sucrose) per pellet of 1 L culture. The mixture was incubated on ice while shaking (200 RPM), and after 1 h osmotic shock was performed using 24 mL of TES buffer diluted 4 times per pellet. Next, MgCl_2_ was added to a final concentration of 10 mM and following cell pellet centrifugation for 30 min at 11,325 g, supernatant (PEs) was collected. Nbs were further purified via immobilized metal affinity chromatography (IMAC) on Ni^2+^ beads (HisPur™ Ni-NTA Resin, Thermo Fischer Scientific) and gel filtration on Superdex™ 75 10/300GL column (Cytiva, Marlborough, MA, USA) in PBS (PBS tablets, Calbiochem, Burlington, MA, USA). The control Nb, R3B23,^[64]^ having no targeting capacity in healthy mice, was produced in the same way.

# Surface plasmon resonance

SPR was performed using a Biacore device (Biacore T200; Cytiva). First, the CM5 sensor chip (Biacore, Series S Sensor Chip CM5; Cytiva) was activated with 1-ethyl-3-(3-dimethylaminopropyl)carbodiimide/N-hydroxysuccinimide (EDC/NHS; Amine Coupling Kit; Cytiva). Subsequently, H, M or CuPAR recombinant proteins (5 µg/mL) were immobilized on separate chips using 10 mM Na-acetate pH 5.0. The SPR measurements were done at 25°C in HEPES buffered saline (HBS, pH 7.4) as a running buffer, with a flow rate of 30 µL/min. For high-throughput off-rate screening with filtered BEs diluted 2 times in HBS (MultiScreenHTS GV Filter Plate, 0.22 µm, Merck), the contact time was set at 120 s, dissociation time at 600 s, and the runs were repeated 5 times. For determination of other kinetic parameters (k_a_, k_d_, K_D_), the Nbs were injected consecutively in three-fold serial dilutions, from 150 to 0.62 nM for 180 s, followed by a dissociation step for 600 s. For both studies, regeneration of 20 s at 30 µL/min using 0.1 M glycine at pH 2.5 followed by a stabilization period of 60 s, was performed. The rate kinetic constants were determined by mathematical fitting using the 1:1 binding with drift and RI2 model proposed by the BIACORE Evaluation Software (Cytiva), and the k_d_/k_a_ ratio was used to determine the equilibrium dissociation constant (K_D_).

# Cell lines and culture conditions

All cells were obtained from the American Type Culture Collection (ATCC, Manassas, VA, USA) and cultured at 37°C, in a humidified atmosphere with 5% CO_2_. After reaching 80% confluency, they were split following dissociation with TrypLE^TM^ Express Enzyme (1X, Gibco). HEK293T cells were cultured in DMEM (Gibco), supplemented with 1% penicillin/streptomycin (10,000 units/mL / 10,000 µg/mL, Gibco), 1% L-glutamine (200 mM, 100X; Gibco) and fetal bovine serum (10% FBS, PAN-Biotech GmbH, Aidenbach, Germany). Mouse and human colorectal adenocarcinoma (respectively MC38 and HT29) and human glioblastoma (U87) cell lines were cultured in media (DMEM for MC38 and U87, and McCoy’s 5A (Modified) Medium (Gibco) for HT29) supplemented as described above. Medium for MC38 and U87 cells was additionally enriched with 1% non-essential amino acids (100X; Gibco) and 1% sodium pyruvate (100 mM, 100X; Gibco).

# Lentiviral vector production and transduction

The production of lentiviral vectors was performed following the established method,^[65]^ and subsequently, HEK293T cells were transfected. The packaging plasmid pCMVΔR8.9 and the VSV.G-encoding plasmid pMD.G were a gift from Dr. Trono (University of Geneva, Geneva, Switzerland). The transfer plasmids pHR’ trip CMV SIN encoding H, M or CuPAR were generated using the Gibson assembly method,^[66]^ based on in silico designed gBlocks from Integrated DNA Technologies, Inc. (IDT, Leuven, Belgium) to contain the cDNA for H, M or CuPAR flanked by 20 base pairs overhangs. The transfer plasmid pHR’ trip CMV eGFP SIN encoding enhanced green fluorescent protein (eGFP) was previously described.^[67]^ The transfer plasmid pDUAL-FLuc/PuroR encoding Firefly Luciferase (FLuc) and puromycin resistance (PuroR) gene was generated using standard cloning techniques.^[68]^

Lentiviral vector transduction of cells was performed as previously described.^[67]^ HEK293T cells were transduced with lentiviral vectors encoding H, M or CuPAR, while MC38 and U87 cells were transduced with lentiviral vectors encoding eGFP and/or FLuc (indicated as GFP+ and FLuc+, respectively). The FLuc+ cells were selected using puromycin (final concentration 1 µg/m L). Following the transduction, cells were characterized with flow cytometry using BD FACSCelesta™ (BD Biosciences, San Jose, CA, USA).

# Flow cytometry

Confluent cell cultures were scraped with a 39 cm Cell Scraper (VWR International B.V., Amsterdam, The Netherlands). Hereafter, 1.5**×**10^5^ cells per well were added to a V-shaped 96-well plate (Greiner Bio-One, Frickenhausen, Germany). Cells were stained in ice cold FACS buffer (PBS, 0.5% BSA, 0.1% NaN_3_) for 1 h with 1 µg/mL of corresponding anti-uPAR APC-labeled (clone REA630-allophycocyanin, Miltenyi Biotec, Leiden, The Netherlands) or PE-labeled (clone VIM5-phycoerythrin, BioLegend) antibody as a positive control, respectively for M or HuPAR. In parallel, to test Nb binding on membrane-associated uPAR, additional cells were incubated for 1 h with 17 nM of the selected H, M or CuPAR binding Nbs and the non-targeting control Nb R3B23, followed by 15 min staining with anti-His-PE (1:50 dilution, clone GG11-8F3.5.1, Miltenyi Biotec). Each incubation step was followed by two washing steps with cold PBS. Finally, the cells were screened with the BD FACSCelesta™ (HTS plate reader) flow cytometer using the BD FACSDiva^TM^ software. Results were analyzed using FlowJo software package (BD Biosciences).

# Thermostability

SYPRO orange gel protein stain (5000X concentrate in DMSO, Life Technologies, Bleiswijk, The Netherlands) at the final concentration of 1X in PBS was added to a reaction mix with 0.2 μg/μL of each tested Nb in PBS until a final volume of 40 μL. In parallel, a blank sample (reaction mix without Nb) was prepared. 12.5 μL of the 40 μL was added to a 96-well (Hard-Shell® 96-Well PCR Plates #HSP9655, Bio-Rad; Hercules, CA, USA) in triplicates. The signal was measured while the plate was run on a thermal cycler (CFX connect™ Real-Time PCR system, Bio-Rad) at temperatures between 25°C and 95°C with steps of 0.5°C/min.

# Technetium-99m-labeling of Nbs

1.5 mL of fresh [^99m^Tc][TcO^-^_4_]-eluate from a ^99m^Tc generator (Drytec, GE Healthcare, Chicago, IL, USA) (around 3700 MBq) was added to a commercial IsoLink kit (Mallinckrodt B.V., Petten, The Netherlands) to reduce [^99m^Tc][TcO^-^_4_] to ^99m^Tc-tricarbonyl ([^99m^Tc][Tc(CO)_3_(H_2_O)_3_]^+^). After boiling for 20 min, 1N HCl (200 μL) was added to neutralize the solution and decompose any residual boranocarbonate. 500 μL of [^99m^Tc][Tc(CO)_3_(H_2_O)_3_]^+^ was added to 1 mg/mL solution of Nb containing a carboxy-terminal His6-tag (50 μL) and incubated for 90 min at 50°C. Hereafter, the solution was loaded on an equilibrated with 10 mL PBS (0.1% Tween) Sephadex G25 disposable column (NAP-5, Cytiva) to remove residual unreacted [^99m^Tc][Tc(CO)_3_(H_2_O)_3_]^+^. The ^99m^Tc-labeled Nb was eluted with 1 mL PBS (0.1% Tween) and the purified Nb was pressed through a Millex filter (0.22 μM; Merck Millipore, Darmstadt, Germany) to eliminate possible aggregates. A radiochemical purity (RCP) of at least 95% is expected and the percentage (%) of radiochemical purity before (RCP_B_) and after (RCP_A_) purification was checked via instant thin layer chromatography (ITLC). 2 μL of ^99m^Tc-labeled Nb was placed on an ITLC strip (Agilent, Santa Clara, CA, USA) approximately 1 cm from the bottom. The bottom of the strip was put in 100% acetone, which was allowed to migrate to the top of the strip. The strip was cut in the middle and the amount of radioactivity on both pieces was measured in a gamma-counter (Veenstra Dose Calibrator, Comecer, Joure, The Netherlands). Furthermore, the reaction radiolabeling yield (RLY), denoted as the ratio of the activity of the final product to the starting activity, and molar activity (A_m_) expressed in GBq/µmol, were determined for all labeled Nbs. These values are presented alongside the radiochemical purity (RCP) in **Table S1**.

# Animal housing and husbandry

All animal experiments were conducted under approval of the ethical commission for animal experimentation (ECD) of the Vrije Universiteit Brussel (projects 19-272-10, 20-272-13, 20-272-14 and 21-272-06). Swiss nude Crl:NU(Ico)-Foxn1^nu^, C57BL/6 WT (Charles River) or C57BL/6 uPAR KO mice (developed by the group of Prof. Peter Carmeliet, KU Leuven,^[60, 61]^ and kindly provided by Prof. Ingrid Struman, ULiège; (in-house breeding, project 20-394-1) were used (4 per group, 6-10 weeks of age; 20-30 g) and housed in individually ventilated cages (3-4 mice per cage) at 19-24°C in 40-60% humidity with a light/dark cycle of 14/10 h. The number of mice allocated to each experiment was determined based on a prior power analysis and mice were randomly assigned to experimental and control groups. Animals had ad libitum access to water and, depending on the injection of fluorescent tracer during the experiment, normal (Safe, Rosenberg, Germany) or low fluorescence food pellets (Teklad 2016, Harlan Laboratories, IN, USA). Following the tumor cell inoculation, mice were visually checked every day for body condition score/body weight loss, physical appearance, and behavior. Mice were weighted and subcutaneous tumors were measured every 2-3 days with a digital caliper. A weight loss of ≥ 20% as compared to the moment of tumor inoculation, subcutaneous tumors with a size ≥ 1500 mm^3^, tumors with severe ulceration, or a severely infected surgical wound (intracranial tumor inoculation), or impacted mobility were considered humane endpoints. All mice were killed via cervical dislocation while either under isoflurane (5% for induction, 2% for maintenance and 1.5 L/min oxygen flow rate) or ketamine/xylazine (intraperitoneal injection, per kg: 100 mg/10 mg) anesthesia.

# In vivo biodistribution of ^99m^Tc-labeled Nbs

Depending on the experiment, different tumor cell types were inoculated (H, M or CuPAR transduced HEK293T cells (10^7^), MC38 cells (10^6^) or U87 cells (2**×**10^6^)) under gas isoflurane anesthesia, in the right flank. After 2-4 weeks, subcutaneous tumors sized between 150-250 mm^3^ were grown. An equivalent of 30–60 MBq radiolabeled Nb (~5 μg) was injected via the tail vein in a volume of 150 μL. Subsequently, mice were anesthetized (intraperitoneal injection, per kg: 100 mg ketamine/10 mg xylazine, 10 min before imaging) and subjected to microSPECT/CT imaging at 1 h post-injection. Imaging was performed using a VECTOR^+^ system (MILabs B.V., Houten, the Netherlands) containing a general-purpose rat/mouse 1.5 mm 75 pinhole collimator. Spiral mode was used to take the scans with 6 bed positions (acquisition time of 200 s per bed position). Two subsets and four iterations were used for image reconstruction, with a voxel size of 0.4 mm in U‐SPECT‐Rec software (MILabs B.V.). The CT scan was made in 1 bed position, with a duration of 146 s at 60 kV and a pixel size of 80 μm. Image analysis was performed using a Medical Image Data Analysis Tool (AMIDE)^[69]^ and 3D images were reconstructed using OsiriX software (OsiriX MD; Pixmeo, Bernex, Switzerland).

After the SPECT/CT scan, mice were killed by cervical dislocation to undergo dissection and organ collection. The radioactivity in each organ and tissues of interest was counted by the gamma counter (2480 WIZARD, Perkin Elmer, Waltham, MA, USA) and expressed as percentage injected activity per gram of tissue (%IA/g), corrected for decay. The biodistribution of each Nb was compared to that of the control Nb.

# Fluorescent labeling of Nbs

4 mg of each of the selected lead targeting Nbs and the control Nb containing a carboxy-terminal His6-tag were separately concentrated into a volume of 500–700 µL using a Vivaspin 2 (5,000 MWCO HY, Sartorius; Stonehouse, UK) at 2,000 g. Each reaction mixture was pH-adjusted by the addition of 100 µL K_2_HPO_4_ (1 M, pH 8.5; VWR International B.V.) to reach a pH of 8.3–8.5. Subsequently, 5 times molar excess of the NHS-ester-activated fluorescent dye s775z (Fluoroprobes, Scottsdale, AZ, USA) was added to the mixtures for an incubation period of 2 h at room temperature. The purification of the labeled Nbs was performed by size-exclusion chromatography (NGCTM Chromatography System, Bio-Rad) using a Superdex™ 75 increase, 10/300 GL column (Cytiva) with PBS as elution buffer at a flow rate of 0.8 mL/min. The labeled Nbs were collected in different fractions by a BioFrac™ fraction collector (Bio-Rad). The concentration of the Nbs (c_Nb_) and the dye (c_dye_) were determined by measuring the absorbance at wavelengths 280 nm and 775 nm, respectively, using a UV-Vis spectrophotometer (NanoDrop™ 2000, Thermo Fisher Scientific) and the use of Lambert-Beer law. For the Nb concentration, a correction factor of 3% was applied to correct for the absorbance of the dye at 280 nm. Finally, the degree of labeling (DOL) was determined by calculating the ratio c_dye_ over c_Nb_. A DOL around 1 was aimed for.

# Spectral characterization of fluorescently-labeled Nbs

Samples of each fluorescent Nb, corresponding to a dye concentration of 1 μM in 1 mL of PBS, and a reference PBS solution were transferred to quartz cuvettes. Excitation and emission (collected at maximal excitation/emission wavelengths, respectively 775 and 794 nm) spectra for the samples were acquired separately using spectrofluorometry (Shimadzu RF-6000, Kyoyto, Japan), every 1 nm between 200 and 900 nm at a scan speed of 2,000 nm/min. The data were normalized to the highest value. The values between 200 and 500 nm were at background level and were excluded from the graphs for clarity.

# In vivo biodistribution of fluorescently-labeled Nbs

Swiss nude Crl:NU(Ico)-Foxn1^nu^ and WT C57BL/6 mice bearing respectively subcutaneous U87 or MC38 tumors were intravenously injected with 100–200 µL containing 2 nmol, based on the concentration of the dye, of Nb15-s775z or Nb13-s775z, respectively and were compared to mice injected with non-targeting control R3B23-s775z. For C57BL/6 mice, the region around the tumor was depilated with a hair removal cream (Veet; RB Healthcare; Liverpool, UK) prior to imaging. Subsequently, dorsal fluorescence imaging was performed 1 h post-injection on isoflurane-anesthetized mice using the NIR fluorescence camera Fluobeam® (Fluoptics, Grenoble, France) in the dark. Immediately after in vivo imaging, mice were killed, and organs of interest were collected for ex vivo imaging. Images were analyzed using ImageJ software (NIH, Bethesda, MD, USA).^[62]^ Regions of interest (ROIs) were determined based on the whole shape of imaged tissues as seen on the white light images.

# Imaging of orthotopic brain tumor using fluorescently-labeled Nbs

Swiss nude Crl:NU(Ico)-Foxn1^nu^ mice were placed in a stereotaxic frame (World Precision Instruments; Hertfordshire, UK) and an incision of 1–1.5 cm ranging from between the eyes to the base of the skull was made. Following the removal of the membrane on the skull with a scalpel, a microdrill was placed above the bregma and was repositioned 2 mm posterior and 2 mm lateral to the right to drill a hole through the skull. Next, 2.5**×**10^5^ of GFP^+^/FLuc^+^ U87 cells resuspended in 5 µL PBS, or 5 µL PBS only (sham surgery) were injected at a depth of 2.5 mm over a period of 5 min with a 10 µL Hamilton syringe (G26s, MICROLITER^TM^ #701N; Hamilton, Reno, NV, USA). Finally, the hole in the skull was sealed off with bone wax and the skin was sutured. The tumor growth in the brain was monitored twice a week, 10 min after intraperitoneal injection of D-Luciferin (150 mg/kg; Promega; Leiden, The Netherlands), by bioluminescence imaging (PhotoIMAGER Optima, Biospacelab; Nesles-La-Vallée, France). Tumors were allowed to grow over a period of 3 weeks. Subsequently, mice were divided into four groups, i.e., mice bearing intracranial tumors injected intravenously with (i) Nb15-s775z, (ii) Nb15-s775z 30 min after injection of 60 times molar excess of unlabeled Nb 15, (iii) control Nb R3B23-s775z and (iv) sham operated mice injected with Nb15-s775z. A dose of 2 nmol, based on the concentration of the dye, was injected per mouse. 1 h after injection of the labeled compound, mice were killed, craniectomy was performed, and fluorescence ex vivo imaging of the brain with Fluobeam® was done in the dark. 10 min prior to killing, D-Luciferin (150 mg/kg) was intraperitoneally injected to enable ex vivo BLI imaging. In addition, GFP imaging was performed using the PhotoIMAGER Optima. Image quantification was performed using ImageJ software. To quantify the fluorescence signal, ROIs were determined based on the GFP images or injection site for sham surgery. Corresponding ROIs were drawn on the healthy left hemisphere to acquire background signal.

# Statistical analyses

Statistical analyses on tumor uptake of radioactively/fluorescently-labeled Nbs were performed using Prism software v.9.4.1. The radioactive Nb uptake in tumor and organs of interest as compared to the control Nb was analyzed using a Kruskal-Wallis test followed by a Dunn’s multiple comparisons test. The tumor uptake of the radioactively-labeled Nb 13 in uPAR KO compared to WT mice, and the fluorescently-labeled Nbs with the associated MFI and TBR analyses in comparison to the control R3B23-s775z were performed using a Mann-Whitney test. Statistical significance was set at p<0.05 (*p<0.05, **p<0.01, ***p<0.001, ****p<0.0001). Descriptive statistics are displayed in the tables as mean ± standard deviation (St. Dev.).

# Supporting Data

**Figure S1**


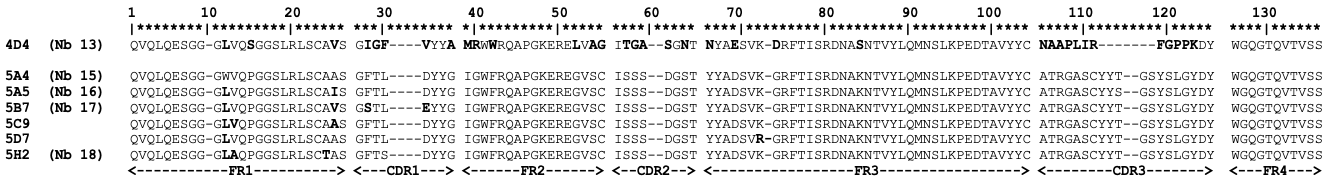


**Figure S1**. Amino acid sequence of the anti-MuPAR Nb 13, and anti HuPAR Nb 15 and its family members, represented according to IMGT standardized criteria. Numbering according to the order of 96-well plates screening is followed by the number of a clone given after selection *in vitro*. Subsequent frameworks and complementarity-determining regions are indicated as FR and CDR, respectively. CDR annotation was performed conforming to the IMGT numbering recommendations.^[70]^ Differences in sequence from the reference sequence (5A4) are indicated in bold.

**Figure S2**

**
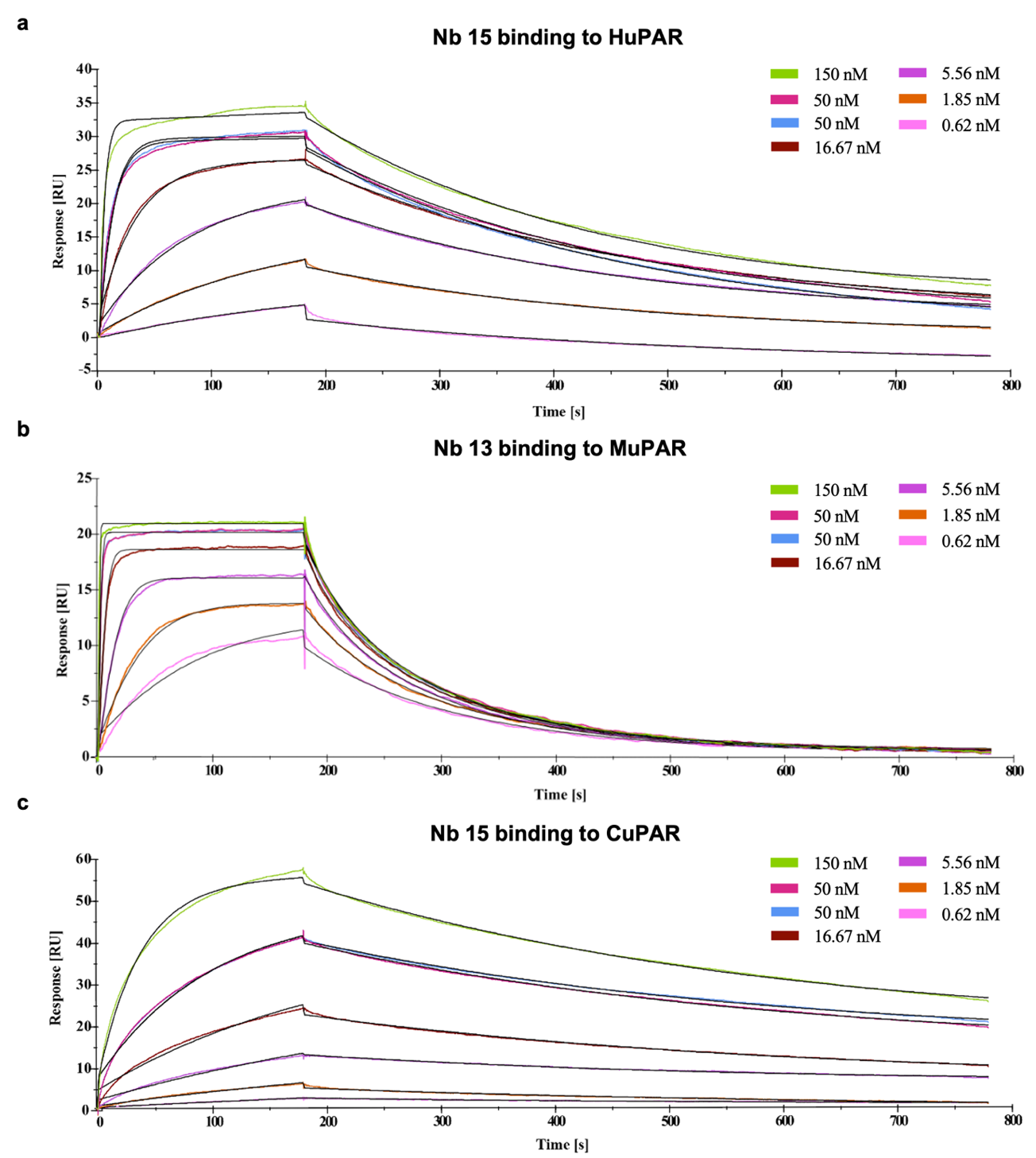
**

**Figure S2.** Sensorgrams of different concentrations (as indicated in the graph) of Nb 15 binding to recombinant H **(A)** and C **(C)** uPAR and Nb 13 binding to recombinant MuPAR **(B)**. Kinetics were measured with a three-fold dilution series of the Nbs (150–0.62 nM). The fitting of the binding curves using the 1:1 binding with drift and RI2 model was applied to calculate kinetic parameters (**Table S1**).

**Table S1**

**Table S1.** Characteristics overview of the nineteen selected uPAR binders divided according to the targeted uPAR homologue (H = human, M = murine, C = canine). All the selected binders were assigned a unique number used consistently in the further description of the experimental progress. The RLY, RCP_B_, RCP_A_, and A_m_ values describe key metrics for radiolabeled compounds, subsequently encompassing parameters such as yield, radiochemical purity both before and after purification, and molar activity. The T_m_ value represents the thermal stability of the Nbs as measured by a thermal shift assay. Kinetic association rates (k_a_), dissociation rates (k_off_, k_d_), and equilibrium constants (K_D_) were measured by SPR. n/a indicates that the clone was excluded from the assay.

| **Human uPAR binders** | | | | | | | | | | | |
| --- | --- | --- | --- | --- | --- | --- | --- | --- | --- | --- | --- |
| **Clone name** | **Nr** | **Prod. yield [mg/l]** | **RLY (%)** | **RCP_B_ (%)** | **RCP_A_ (%)** | **A_m_ [GBq/µmol]** | **T_m_ [°C]** | **k_off_  [s^-1^]**  **(Hu)** | **k_a_  [M^-1^×s^-1^] (Hu)** | **k_d_  [s^-1^]**  **(Hu)** | **K_D_ [M]**  **(Hu)** |
| **1A4** | **1** | 1.85 | n/a | n/a | n/a | n/a | 64.8 | 5.1E-03 | 3.5E+05 | 6.2E-03 | 1.8E-08 |
| **1A6** | **2** | 6.96 | n/a | n/a | n/a | n/a | 63.5 | 1.7E-01 | 1.7E+06 | 2.3E-01 | 1.4E-07 |
| **1C8** | **3** | 3.34 | 67 | 98 | 100 | 157.83 | 73.6 | 8.9E-03 | 1.2E+06 | 7.5E-03 | 6.0E-09 |
| **1D8** | **4** | 4.14 | 48 | 96 | 99 | 178.64 | 60.4 | 4.3E-03 | 1.2E+06 | 5.2E-03 | 6.0E-09 |
| **2A4** | **5** | 0.53 | n/a | n/a | n/a | n/a | n/a | 6.2E-03 | 8.6E+05 | 7.3E-03 | 8.4E-09 |
| **2C6** | **6** | 2.03 | n/a | n/a | n/a | n/a | n/a | 6.4E-03 | 4.6E+05 | 7.2E-03 | 1.6E-08 |
| **2C9** | **7** | 7.29 | n/a | n/a | n/a | n/a | 66.8 | 1.3E-03 | 1.1E+06 | 2.2E-01 | 2.0E-07 |
| **2D1** | **8** | 4.80 | n/a | n/a | n/a | n/a | 59.5 | 8.6E-04 | 6.1E+04 | 1.0E-03 | 1.7E-08 |
| **2D4** | **19** | 1.29 | n/a | n/a | n/a | n/a | 67.9 | 9.9E-03 | 4.3E+05 | 3.5E-03 | 8.2E-09 |
| **5A4** | **15** | 2.30 | 55 | 96 | 100 | 163.33 | 61.2 | 4.2E-03 | 1.6E+06 | 3.4E-03 | 2.1E-09 |
| **5A5** | **16** | 2.14 | 54 | 95 | 100 | 162.83 | 66.5 | 2.7E-03 | 5.0E+05 | 1.5E-03 | 3.0E-09 |
| **5B7** | **17** | 4.00 | 56 | 89 | 99 | 162.32 | 66.3 | 2.6E-03 | 8.6E+05 | 1.9E-03 | 2.3E-09 |
| **5H2** | **18** | 3.63 | 54 | 96 | 100 | 162.25 | 62.1 | 3.1E-03 | 8.4E+05 | 4.0E-03 | 4.7E-09 |
| **Mouse uPAR binders** | | | | | | | | | | | |
| **Clone name** | **Nr** | **Prod. yield [mg/l]** | **RLY (%)** | **RCPB (%)** | **RCPA (%)** | **A_m_ [GBq/µmol]** | **T_m_ [°C]** | **k_off_  [s^-1^]**  **(Hu)** | **k_a_  [M^-1^×s^-1^] (Hu)** | **k_d_  [s^-1^]**  **(Hu)** | **K_D_ [M]**  **(Hu)** |
| **3B10** | **9** | 4.74 | n/a | n/a | n/a | n/a | 64.3 | 4.6E-03 | 2.57E+05 | 4.66E-03 | 1.8E-08 |
| **4B8** | **10** | 7.00 | 51 | 94 | 99 | 148.09 | 65.4 | 2.6E-06 | 2.39E+05 | 2.64E-03 | 1.1E-08 |
| **4B10** | **11** | 6.30 | 55 | 95 | 100 | 149.20 | 69.4 | 3.4E-03 | 1.69E+06 | 7.30E-03 | 4.3E-09 |
| **4C3** | **12** | 8.40 | n/a | n/a | n/a | n/a | 69.1 | 2.3E-01 | 1.89E+05 | 5.41E-02 | 2.9E-07 |
| **4D4** | **13** | 7.40 | 55 | 96 | 99 | 150.64 | 72.4 | 7.5E-03 | 1.76E+07 | 1.45E-02 | 8.3E-10 |
| **4D11** | **14** | 8.60 | 52 | 96 | 99 | 148.63 | 62.4 | 1.9E-03 | 1.11E+05 | 1.14E-03 | 1.0E-08 |
| **Canine uPAR binders** | | | | | | | | | | | |
| **Clone name** | **Nr** | **Prod. yield [mg/l]** | **RLY (%)** | **RCPB (%)** | **RCPA (%)** | **A_m_ [GBq/µmol]** | **T_m_ [°C]** | **k_off_  [s^-1^]**  **(Hu)** | **k_a_  [M^-1^×s^-1^] (Hu)** | **k_d_  [s^-1^]**  **(Hu)** | **K_D_ [M]**  **(Hu)** |
| **5A4** | **15** | 2.30 | 55 | 96 | 100 | 163.33 | 61.2 | 6.5E-03 | 1.7E+05 | 2.0E-03 | 1.2E-08 |
| **5A5** | **16** | 2.14 | 54 | 95 | 100 | 162.83 | 66.5 | 1.0E-02 | 8.6E+04 | 3.1E-03 | 3.6E-08 |
| **5B7** | **17** | 4.00 | 56 | 89 | 99 | 162.32 | 66.3 | 7.3E-03 | 1.2E+05 | 1.9E-03 | 1.7E-08 |
| **5H2** | **18** | 3.63 | 54 | 96 | 100 | 162.25 | 62.1 | 9.2E-03 | 1.0E+05 | 3.5E-03 | 3.4E-08 |

**Table S2**

**Table S2**. *Ex vivo* biodistribution of [^99m^Tc]Tc labeled-Nbs in Swiss nude mice bearing a subcutaneous (H, M or C) uPAR-expressing HEK tumor. Adjusted P values were calculated based on the comparisons with control Nb.

| **Human uPAR binders** | | | | | | | | | | | | | |
| --- | --- | --- | --- | --- | --- | --- | --- | --- | --- | --- | --- | --- | --- |
| **Clone nr**  **Organ** | **Ctrl Nb** | **Nb 3** | | **Nb 4** | | **Nb 15** | | **Nb 16** | | **Nb 17** | | **Nb 18** | |
|  | Mean±St. Dev. (%IA/g organ) | Mean±St. Dev. (%IA/g organ) | Adjusted P Value | Mean±St. Dev. (%IA/g organ) | Adjusted P Value | Mean±St. Dev. (%IA/g organ) | Adjusted P Value | Mean±St. Dev. (%IA/g organ) | Adjusted P Value | Mean±St. Dev. (%IA/g organ) | Adjusted P Value | Mean±St. Dev. (%IA/g organ) | Adjusted  P Value |
| **Heart** | 0.43±0.16 | 0.26±0.05 | ns | 0.64±0.11 | ns | 0.52±0.33 | ns | 1.09±0.15 | ns | 0.71±0.19 | ns | 0.24±0.09 | ns |
| **Spleen** | 0.27±0.05 | 0.92±0.51 | ns | 0.66±0.07 | ns | 0.78±0.33 | ns | 3.04±1.01 | 0.001 | 1.58±0.76 | 0.017 | 0.72±0.16 | ns |
| **Pancreas** | 0.22±0.04 | 0.32±0.06 | ns | 0.36±0.07 | ns | 0.43±0.23 | ns | 0.94±0.28 | 0.001 | 0.46±0.08 | 0.013 | 0.34±0.02 | ns |
| **Stomach** | 0.66±0.64 | 0.28±0.07 | ns | 0.83±0.16 | ns | 0.76±0.49 | ns | 2.24±0.50 | ns | 0.71±0.18 | ns | 0.33±0.07 | ns |
| **Small intestine** | 0.28±0.08 | 0.29±0.06 | ns | 0.68±0.18 | ns | 0.49±0.16 | ns | 1.63±0.18 | 0.006 | 0.99±0.49 | 0.028 | 0.34±0.06 | ns |
| **Large intestine** | 0.31±0.09 | 0.28±0.03 | ns | 0.84±0.09 | ns | 0.47±0.28 | ns | 1.15±0.09 | 0.007 | 0.99±0.36 | ns | 0.38±0.04 | ns |
| **Bone** | 0.20±0.02 | 0.21±0.07 | ns | 0.32±0.05 | ns | 0.53 ±0.59 | ns | 0.66±0.13 | 0.029 | 0.35±0.12 | ns | 0.17±0.03 | ns |
| **Lymph nodes** | 0.30±0.11 | 0.29±0.10 | ns | 0.59±0.36 | ns | 0.40±0.13 | ns | 1.42±0.89 | ns | 0.66±0.29 | ns | 0.37±0.10 | ns |
| **Kidneys** | 156±25 | 110±33 | ns | 328±39 | 0.003 | 119±16 | ns | 275±35 | 0.049 | 193±28 | ns | 197±42 | ns |
| **Lungs** | 0.68±0.26 | 0.56±0.15 | ns | 1.57±0.35 | ns | 0.98±0.62 | ns | 2.36±0.81 | ns | 1.06±0.28 | ns | 0.52±0.15 | ns |
| **Liver** | 0.77±0.20 | 2.66±1.31 | ns | 2.07±0.42 | ns | 1.81±0.26 | ns | 13.56±1.07 | 0.001 | 3.02±1.03 | 0.015 | 2.00±0.36 | ns |
| **Muscle** | 0.15±0.05 | 0.12±0.03 | ns | 0.31±0.05 | ns | 0.41±0.56 | ns | 0.38±0.07 | ns | 0.23±0.08 | ns | 0.11±0.01 | ns |
| **Blood** | 1.03±0.35 | 0.46±0.12 | ns | 1.16±0.18 | ns | 0.68±0.37 | ns | 3.58±0.07 | ns | 1.78±0.51 | ns | 0.38±0.14 | ns |
| **Tumor** | 0.20±0.08 | 0.49±0.12 | ns | 1.28±0.27 | ns | 1.98±0.79 | 0.004 | 1.96±0.13 | 0.003 | 1.64±0.37 | 0.017 | 1.15±0.16 | ns |
| **Mouse uPAR binders** | | | | | | | | | |  |  |  |  |
| **Clone nr**  **Organ** | **Ctrl Nb** | **Nb 10** | | **Nb 11** | | **Nb 13** | | **Nb 14** | |  |  |  |  |
|  | Mean±St. Dev. (%IA/g organ) | Mean±St. Dev. (%IA/g organ) | Adjusted P Value | Mean±St. Dev. (%IA/g organ) | Adjusted P Value | Mean±St. Dev. (%IA/g organ) | Adjusted P Value | Mean±St. Dev. (%IA/g organ) | Adjusted P Value |  |  |  |  |
| **Heart** | 0.22±0.07 | 0.21±0.05 | ns | 1.01±0.20 | 0.004 | 0.64±0.10 | ns | 0.48±0.12 | ns |  |  |  |  |
| **Spleen** | 0.15±0.01 | 0.20±0.04 | ns | 6.02±0.19 | 0.001 | 3.77±0.19 | 0.023 | 1.79±1.70 | ns |  |  |  |  |
| **Pancreas** | 0.13±0.04 | 0.14±0.03 | ns | 0.90±0.39 | 0.004 | 0.62±0.31 | 0.048 | 0.30±0.15 | ns |  |  |  |  |
| **Stomach** | 0.24±0.06 | 0.36±0.05 | ns | 1.54±0.12 | 0.002 | 0.84±0.11 | ns | 0.70±0.40 | ns |  |  |  |  |
| **Small intestine** | 0.17±0.02 | 0.31±0.07 | ns | 1.712±0.10 | 0.001 | 0.92±0.15 | 0.048 | 0.68±0.35 | ns |  |  |  |  |
| **Large intestine** | 0.24±0.08 | 0.27±0.02 | ns | 1.84±0.28 | 0.001 | 1.00±0.20 | ns | 0.76±0.41 | ns |  |  |  |  |
| **Bone** | 0.14±0.02 | 0.13±0.05 | ns | 1.50±0.27 | 0.004 | 0.87±0.11 | ns | 0.43±0.15 | ns |  |  |  |  |
| **Lymph nodes** | 0.24±0.06 | 0.34±0.19 | ns | 1.34±0.96 | ns | 1.17±1.01 | ns | 0.59±0.43 | ns |  |  |  |  |
| **Kidneys** | 68±8 | 65±7 | ns | 158±48 | 0.002 | 145±36 | 0.003 | 134±49 | 0.017 |  |  |  |  |
| **Lungs** | 0.45±0.05 | 0.60±0.06 | ns | 8.83±1.13 | 0.001 | 4.28±0.54 | ns | 2.89±2.07 | ns |  |  |  |  |
| **Liver** | 0.31±0.02 | 0.77±0.17 | ns | 1.31±0.11 | 0.028 | 1.94±0.85 | 0.008 | 1.34±0.25 | 0.048 |  |  |  |  |
| **Muscle** | 0.11±0.02 | 0.14±0.04 | ns | 0.33±0.05 | 0.007 | 0.27±0.03 | ns | 0.27±0.15 | ns |  |  |  |  |
| **Blood** | 0.50±0.07 | 0.52±0.12 | ns | 0.56±0.11 | ns | 0.73±0.03 | 0.033 | 0.74±0.19 | ns |  |  |  |  |
| **Tumor** | 0.13±0.07 | 0.38±0.12 | ns | 2.46±0.23 | 0.004 | 2.17±0.74 | 0.008 | 1.17±0.57 | ns |  |  |  |  |
| **Canine uPAR binders** | | | | | |  |  |  |  |  |  |  |  |
| **Clone nr**  **Organ** | **Ctrl Nb** | **Nb 15** | | **Nb 17** | |  |  |  |  |  |  |  |  |
|  | Mean±St. Dev. (%IA/g organ) | Mean±St. Dev. (%IA/g organ) | Adjusted P Value | Mean±St. Dev. (%IA/g organ) | Adjusted P Value |  |  |  |  |  |  |  |  |
| **Heart** | 0.19±0.05 | 0.54±0.28 | ns | 0.70±0.25 | 0.0057 |  |  |  |  |  |  |  |  |
| **Spleen** | 0.16±0.04 | 0.72±0.10 | ns | 2.12±0.63 | 0.0023 |  |  |  |  |  |  |  |  |
| **Pancreas** | 0.13±0.05 | 0.36±0.07 | ns | 0.58±0.13 | 0.0022 |  |  |  |  |  |  |  |  |
| **Stomach** | 0.20±0.06 | 0.70±0.14 | 0.020 | 0.84±0.22 | 0.0045 |  |  |  |  |  |  |  |  |
| **Small intestine** | 0.17±0.04 | 0.92±0.41 | 0.016 | 1.06±0.40 | 0.0005 |  |  |  |  |  |  |  |  |
| **Large intestine** | 0.27±0.19 | 0.71±0.57 | ns | 0.67±0.17 | ns |  |  |  |  |  |  |  |  |
| **Bone** | 0.16±0.01 | 0.34±0.13 | ns | 0.51±0.10 | 0.0165 |  |  |  |  |  |  |  |  |
| **Lymph nodes** | 0.30±0.06 | 0.40±0.17 | ns | 0.98±0.18 | 0.0255 |  |  |  |  |  |  |  |  |
| **Kidneys** | 66±10 | 127±30 | ns | 208±22 | 0.0002 |  |  |  |  |  |  |  |  |
| **Lungs** | 0.42±0.08 | 0.86±0.30 | ns | 1.38±0.33 | 0.0049 |  |  |  |  |  |  |  |  |
| **Liver** | 0.28±0.05 | 1.85±0.20 | ns | 6.10±1.14 | 0.0008 |  |  |  |  |  |  |  |  |
| **Muscle** | 0.12±0.02 | 0.21±0.13 | ns | 0.30±0.08 | 0.0213 |  |  |  |  |  |  |  |  |
| **Blood** | 0.51±0.15 | 0.82±0.27 | ns | 2.21±0.68 | 0.0180 |  |  |  |  |  |  |  |  |
| **Tumor** | 0.18±0.04 | 1.46±0.03 | 0.012 | 1.70±0.31 | 0.0010 |  |  |  |  |  |  |  |  |

**Table S3**

**Table S3.** *Ex vivo* biodistribution of [^99m^Tc]Tc-Nbs in Swiss nude mice bearing a subcutaneous U87 tumor. Adjusted P-values were calculated based on the comparisons with control Nb.

| **Clone nr**  **Organ** | **Ctrl Nb** | **Nb 4** | | **Nb 15** | |
| --- | --- | --- | --- | --- | --- |
|  | Mean±St. Dev. (%IA/g organ) | Mean±St. Dev. (%IA/g organ) | Adjusted P Value | Mean±St. Dev. (%IA/g organ) | Adjusted P Value |
| **Heart** | 0.29±0.12 | 0.44±0.11 | ns | 0.30±0.09 | ns |
| **Lungs** | 0.51±0.17 | 1.11±0.21 | 0.016 | 0.60±0.11 | ns |
| **Liver** | 0.53±0.18 | 1.43±0.49 | 0.029 | 0.83±0.05 | ns |
| **Spleen** | 0.22±0.07 | 0.48±0.14 | 0.048 | 0.33±0.03 | ns |
| **Pancreas** | 0.23±0.13 | 0.31±0.10 | ns | 0.31±0.08 | ns |
| **Kidneys** | 284±68 | 152±73 | 0.004 | 200±15 | ns |
| **Stomach** | 0.95±0.56 | 0.94±0.43 | ns | 0.59±0.19 | ns |
| **Small intestine** | 0.32±0.05 | 0.48±0.12 | ns | 0.36±0.09 | ns |
| **Large intestine** | 0.31±0.11 | 0.67±0.24 | ns | 0.37±0.08 | ns |
| **Muscle** | 0.15±0.06 | 0.09±0.03 | ns | 0.18±0.03 | ns |
| **Bone** | 0.15±0.07 | 0.22±0.17 | ns | 0.15±0.04 | ns |
| **Blood** | 0.54±0.23 | 1.00±0.65 | ns | 0.47±0.15 | ns |
| **Lymph nodes** | 0.75±0.30 | 0.94±0.41 | ns | 0.74±0.29 | ns |
| **Tumor** | 0.30±0.12 | 0.67±0.26 | ns | 3.01±0.60 | 0.003 |

**Table S4**

**Table S4.** *Ex vivo* biodistribution of [^99m^Tc]Tc-Nbs in WT and uPAR KO C57BL/6 mice bearing a subcutaneous syngeneic MC38 tumor. Adjusted P values were calculated based on the comparisons with control Nb. P values were calculated based on the comparison between WT and uPAR KO mice.

| **C57BL/6 WT** | | | | | | | | | | |
| --- | --- | --- | --- | --- | --- | --- | --- | --- | --- | --- |
| **Clone nr**  **Organ** | | **Ctrl Nb** | **Nb 11** | | | **Nb 13** | | | |  |
|  |  | Mean±St. Dev. (%IA/g organ) | Mean±St. Dev. (%IA/g organ) | | Adjusted P value | Mean±St. Dev. (%IA/g organ) | | | Adjusted P value |  |
| **Heart** | | 0.33±0.12 | 0.89±0.10 | | 0.007 | 0.74±0.05 | | | ns |  |
| **Lungs** | | 1.33±1.62 | 11.43±1.37 | | 0.006 | 7.78±1.02 | | | ns |  |
| **Liver** | | 0.57±0.17 | 1.52±0.24 | | ns | 3.03±0.47 | | | 0.006 |  |
| **Spleen** | | 0.23±0.06 | 5.87±0.46 | | 0.006 | 4.37±0.53 | | | ns |  |
| **Pancreas** | | 0.20±0.06 | 1.10±0.20 | | 0.006 | 0.72±0.03 | | | ns |  |
| **Kidneys** | | 214±44 | 158±14 | | 0.016 | 242±25 | | | ns |  |
| **Stomach** | | 0.63±0.19 | 1.64±0.18 | | 0.021 | 1.49±0.24 | | | ns |  |
| **Small intestine** | | 0.28±0.08 | 1.80±0.12 | | 0.008 | 1.37±0.37 | | | ns |  |
| **Large intestine** | | 0.33±0.07 | 1.94±0.75 | | 0.016 | 1.86±0.54 | | | ns |  |
| **Muscle** | | 0.13±0.01 | 0.33±0.03 | | 0.009 | 0.24±0.08 | | | ns |  |
| **Bone** | | 0.17±0.06 | 1.28±0.21 | | 0.015 | 1.03±0.25 | | | ns |  |
| **Blood** | | 0.51±0.14 | 0.40±0.05 | | ns | 0.54±0.03 | | | ns |  |
| **Lymph nodes** | | 0.43±0.07 | 1.54±1.01 | | 0.028 | 1.20±0.67 | | | ns |  |
| **Tumor** | | 0.41±0.07 | 4.93±0.11 | | ns | 5.55±0.80 | | | 0.030 |  |
| **C57BL/6 uPAR KO** | | | |  | | |  |  |  |  |
| **Clone nr**  **Organ** | **Nb 13 in WT mouse** | | | **Nb 13 in KO mouse** | | | |  |  |  |
|  | Mean±St. Dev. (%IA/g organ) | | | Mean±St. Dev. (%IA/g organ) | | | P value |  |  |  |
| **Heart** | 0.74±0.05 | | | 0.24±0.08 | | | 0.029 |  |  |  |
| **Lungs** | 7.78±1.02 | | | 0.89±0.58 | | | 0.029 |  |  |  |
| **Liver** | 3.03±0.47 | | | 1.63±0.46 | | | 0.029 |  |  |  |
| **Spleen** | 4.37±0.53 | | | 0.38±0.14 | | | 0.029 |  |  |  |
| **Pancreas** | 0.72±0.03 | | | 0.15±0.02 | | | 0.029 |  |  |  |
| **Kidneys** | 242±25 | | | 209±33 | | | 0.041 |  |  |  |
| **Stomach** | 1.49±0.24 | | | 0.39±0.24 | | | 0.029 |  |  |  |
| **Small intestine** | 1.37±0.37 | | | 0.34±0.17 | | | 0.029 |  |  |  |
| **Large intestine** | 1.86±0.54 | | | 0.32±0.08 | | | 0.029 |  |  |  |
| **Muscle** | 0.24±0.08 | | | 0.09±0.01 | | | 0.029 |  |  |  |
| **Bone** | 1.03±0.25 | | | 0.16±0.06 | | | 0.029 |  |  |  |
| **Blood** | 0.54±0.03 | | | 0.57±0.19 | | | ns |  |  |  |
| **Lymph nodes** | 1.20±0.67 | | | 0.28±0.09 | | | ns |  |  |  |
| **Tumor** | 5.55±0.80 | | | 1.71±0.53 | | | 0.029 |  |  |  |

Received: ((will be filled in by the editorial staff))
Revised: ((will be filled in by the editorial staff))
Published online: ((will be filled in by the editorial staff))

1. Wyld L, Audisio RA, and Poston GJ (**2015**) The evolution of cancer surgery and future perspectives. Nat Rev Clin Oncol 12:115–124

2. Mieog JSD, Achterberg FB, Zlitni A, Hutteman M, Burggraaf J, Swijnenburg R-J, Gioux S, and Vahrmeijer AL (**2022**) Fundamentals and developments in fluorescence-guided cancer surgery. Nat Rev Clin Oncol 19:9–22

3. Schouw HM, Huisman LA, Janssen YF, Slart RHJA, Borra RJH, Willemsen ATM, Brouwers AH, Dijl JM van, Dierckx RA, Dam GM van, Szymanski W, Boersma HH, and Kruijff S (**2021**) Targeted optical fluorescence imaging: a meta-narrative review and future perspectives. Eur J Nucl Med Mol Imaging 48:4272–4292

4. FDA IC-GREEN, https://www.accessdata.fda.gov/drugsatfda_docs/label/2015/011525s027lbl.pdf

5. Rossi G, Tarasconi A, Baiocchi G, De’angelis GL, Gaiani F, Mario F Di, Catena F, and Dalla Valle R (**2018**) Fluorescence guided surgery in liver tumors: Applications and advantages. Acta Biomed 89:135–140

6. Wishart GC, Loh SW, Jones L, and Benson JR (**2012**) A feasibility study (ICG-10) of indocyanine green (ICG) fluorescence mapping for sentinel lymph node detection in early breast cancer. Eur J Surg Oncol 38:651–656

7. Jafari MD, Lee KH, Halabi WJ, Mills SD, Carmichael JC, Stamos MJ, and Pigazzi A (**2013**) The use of indocyanine green fluorescence to assess anastomotic perfusion during robotic assisted laparoscopic rectal surgery. Surg Endosc 27:3003–3008

8. Mothes H, Friedel R, Simon M, Markgraf E, and Bach O (**2004**) Indocyanine-Green Fluorescence Video Angiography Used Clinically to Evaluate Tissue Perfusion in Microsurgery. 13–17

9. Gliolan | European Medicines Agency, https://www.ema.europa.eu/en/medicines/human/EPAR/gliolan

10. Tanyi JL, Randall LM, Chambers SK, Butler KA, Winer IS, Langstraat CL, Han ES, Vahrmeijer AL, Chon HS, Morgan MA, Powell MA, Tseng JH, Lopez AS, and Wenham RM (**2022**) A Phase III Study of Pafolacianine Injection (OTL38) for Intraoperative Imaging of Folate Receptor-Positive Ovarian Cancer (Study 006). J Clin Oncol 41:276–284

11. Kennedy GT, Azari FS, Bernstein E, Nadeem B, Chang A, Segil A, Sullivan N, Encarnado E, Desphande C, Kucharczuk JC, Leonard K, Low PS, Chen S, Criton A, and Singhal S (**2022**) Targeted detection of cancer cells during biopsy allows real-time diagnosis of pulmonary nodules. Eur J Nucl Med Mol Imaging 49:4194–4204

12. CYTALUX® for lung and ovarian cancer surgery, https://cytalux.com/

13. Hernandez Vargas S, Lin C, Tran Cao HS, Ikoma N, AghaAmiri S, Ghosh SC, Uselmann AJ, and Azhdarinia A (**2021**) Receptor-Targeted Fluorescence-Guided Surgery With Low Molecular Weight Agents. Front Oncol 11:1–15

14. Hernot S, Manen L van, Debie P, Mieog JSD, and Vahrmeijer AL (**2019**) Latest developments in molecular tracers for fluorescence image-guided cancer surgery. Lancet Oncol 20:e354–e367

15. Debie P and Hernot S (**2019**) Emerging Fluorescent Molecular Tracers to Guide Intra-Operative Surgical Decision-Making. Front Pharmacol 10:510

16. Smith HW and Marshall CJ (**2010**) Regulation of cell signalling by uPAR. Nat Rev Mol Cell Biol 11:23–36

17. Boonstra MC, Prakash J, Velde CJH Van De, Mesker WE, Kuppen PJK, Vahrmeijer AL, and Sier CFM (**2015**) Stromal targets for fluorescent-guided oncologic surgery. Front Oncol 5:1–8

18. Geus SWL de, Boogerd LSF, Swijnenburg RJ, Mieog JSD, Tummers WSFJ, Prevoo HAJM, Sier CFM, Morreau H, Bonsing BA, Velde CJH van de, Vahrmeijer AL, and Kuppen PJK (**2016**) Selecting Tumor-Specific Molecular Targets in Pancreatic Adenocarcinoma: Paving the Way for Image-Guided Pancreatic Surgery. Mol Imaging Biol 18:807–819

19. Christensen A, Grønhøj C, Jensen JS, Lelkaitis G, Kiss K, Juhl K, Charabi BW, Mortensen J, Kjær A, and Buchwald C Von (**2022**) Expression patterns of uPAR, TF and EGFR and their potential as targets for molecular imaging in oropharyngeal squamous cell carcinoma. Oncol Rep 48

20. Metrangolo V, Ploug M, and Engelholm LH (**2021**) The urokinase receptor (uPAR) as a “trojan horse” in targeted cancer therapy: Challenges and opportunities. Cancers (Basel) 13

21. Zhai BT, Tian H, Sun J, Zou JB, Zhang XF, Cheng JX, Shi YJ, Fan Y, and Guo DY (**2022**) Urokinase-type plasminogen activator receptor (uPAR) as a therapeutic target in cancer. J Transl Med 20:1–24

22. K. Lund I, Illemann M, Thurison T, J. Christensen I, and Hoyer-Hansen G (**2011**) uPAR as Anti-Cancer Target: Evaluation of Biomarker Potential, Histological Localization, and Antibody-Based Therapy. Curr Drug Targets 12:1744–1760

23. Mahmood N, Mihalcioiu C, and Rabbani SA (**2018**) Multifaceted role of the urokinase-type plasminogen activator (uPA) and its receptor (uPAR): Diagnostic, prognostic, and therapeutic applications. Front Oncol 8

24. Yang L, Sajja HK, Cao Z, Qian W, Bender L, Marcus AI, Lipowska M, Wood WC, and Andrew Wang Y (**2014**) uPAR-targeted optical imaging contrasts as theranostic agents for tumor margin detection. Theranostics 4:106–118

25. Baart VM, Houvast RD, Geus-Oei LF De, Quax PHA, Kuppen PJK, Vahrmeijer AL, and Sier CFM (**2020**) Molecular imaging of the urokinase plasminogen activator receptor: opportunities beyond cancer. EJNMMI Res 10

26. Ding F, Chen S, Zhang W, Tu Y, and Sun Y (**2017**) UPAR targeted molecular imaging of cancers with small molecule-based probes. Bioorganic Med Chem 25:5179–5184

27. Muyldermans S (**2013**) Nanobodies: Natural Single-Domain Antibodies. Annu Rev Biochem 82:775–797

28. Debie P, Devoogdt N, and Hernot S (**2019**) Targeted Nanobody-Based Molecular Tracers for Nuclear Imaging and Image-Guided Surgery. 8:12

29. Muyldermans S (**2021**) Applications of Nanobodies. Annu Rev Anim Biosci 9:401–421

30. Chakravarty R, Goel S, and Cai W (**2014**) Nanobody: The “magic bullet” for molecular imaging? Theranostics 4:386–398

31. PLAUR protein expression summary - The Human Protein Atlas, https://www.proteinatlas.org/ENSG00000011422-PLAUR

32. Solberg H, Ploug M, Høyer-Hansen G, Nielsen BS, and Lund LR (**2001**) The murine receptor for urokinase-type plasminogen activator is primarily expressed in tissues actively undergoing remodeling. J Histochem Cytochem 49:237–246

33. Valle NCH do, Janssen S, Stroet MCM, Pollenus S, Block S Van den, Devoogdt N, Debacker JM, Hernot S, and Rooster H De (**2023**) Safety assessment of fluorescently labeled anti-EGFR Nanobodies in healthy dogs. Front Pharmacol 14:1–10

34. Carlsen EA, Loft M, Loft A, Berthelsen AK, Langer SW, Knigge U, and Kjaer A (**2022**) Prospective Phase II Trial of Prognostication by 68 Ga-NOTA-AE105 uPAR PET in Patients with Neuroendocrine Neoplasms: Implications for uPAR-Targeted Therapy . J Nucl Med 63:1371–1377

35. Risør LM, Clausen MM, Ujmajuridze Z, Farhadi M, Andersen KF, Loft A, Friborg J, and Kjaer A (**2022**) Prognostic Value of Urokinase-Type Plasminogen Activator Receptor PET/CT in Head and Neck Squamous Cell Carcinomas and Comparison with 18F-FDG PET/CT: A Single-Center Prospective Study. J Nucl Med 63:1169–1176

36. Fosbøl MØ, Mortensen J, Petersen PM, Loft A, Madsen J, and Kjaer A (**2021**) uPAR PET/CT for Prognostication and Response Assessment in Patients with Metastatic Castration-Resistant Prostate Cancer Undergoing Radium-223 Therapy: A Prospective Phase II Study. 11:1087

37. Xavier C, Blykers A, Laoui D, Bolli E, Vaneyken I, Bridoux J, Baudhuin H, Raes G, Everaert H, Movahedi K, Ginderachter JA Van, Devoogdt N, Caveliers V, Lahoutte T, and Keyaerts M (**2019**) Clinical Translation of [68Ga]Ga-NOTA-anti-MMR-sdAb for PET/CT Imaging of Protumorigenic Macrophages. Mol Imaging Biol 21:898–906

38. Bridoux J, Neyt S, Debie P, Descamps B, Devoogdt N, Cleeren F, Bormans G, Broisat A, Caveliers V, Xavier C, Vanhove C, and Hernot S (**2020**) Improved detection of molecular markers of atherosclerotic plaques using sub-millimeter PET imaging. 25:1–10

39. Debie P, Quathem J Van, Hansen I, Bala G, Massa S, Devoogdt N, Xavier C, and Hernot S (**2017**) Effect of dye and conjugation chemistry on the biodistribution profile of near-infrared-labeled nanobodies as tracers for image-guided surgery. Mol Pharm 14:1145–1153

40. Bunschoten A, Willigen DM Van, Buckle T, Berg NS Van Den, Welling MM, Spa SJ, Wester HJ, and Leeuwen FWB Van (**2016**) Tailoring Fluorescent Dyes to Optimize a Hybrid RGD-Tracer. Bioconjug Chem 27:1253–1258

41. Usama SM, Thapaliya ER, Luciano MP, and Schnermann MJ (**2021**) Not so innocent: Impact of fluorophore chemistry on the in vivo properties of bioconjugates. Curr Opin Chem Biol 63:38–45

42. Declerck NB, Declercq S, Debie P, Ville J Du, Mateusiak L, Stroet M, Tariq Akhtar R, Luciano M, Dong-Hao L, Schnermann MJ, Smith B, and Hernot S (**2023**) The role of the near-infrared fluorescent dye on the pharmacokinetic behavior of fluorescent nanobody-based tracers for intra-operative cancer imaging.

43. Li DH, Schreiber CL, and Smith BD (**2020**) Sterically Shielded Heptamethine Cyanine Dyes for Bioconjugation and High Performance Near-Infrared Fluorescence Imaging. Angew Chem Int Ed Engl 59:12154–12161

44. Weller M, Bent M van den, Preusser M, Rhun E Le, Tonn JC, Minniti G, Bendszus M, Balana C, Chinot O, Dirven L, French P, Hegi ME, Jakola AS, Platten M, Roth P, Rudà R, Short S, Smits M, Taphoorn MJB, et al (**2021**) EANO guidelines on the diagnosis and treatment of diffuse gliomas of adulthood. Nat Rev Clin Oncol 18:170–186

45. Sanai N and Berger MS (**2018**) Surgical oncology for gliomas: The state of the art. Nat Rev Clin Oncol 15:112–125

46. Hadjipanayis CG, Fiske CT, Jones TF, Warkentin J, Shepherd BE, Maruri F, and Sterling TR (**2015**) What is the Surgical Benefit of Utilizing 5-ALA for Fluroscence-Guided-Surgery of Malignant Gliomas? Neurosurgery 143:951–959

47. Skjøth-Rasmussen J, Azam A, Larsen CC, Scheie D, Juhl K, and Kjaer A (**2021**) A new uPAR-targeting fluorescent probe for optical guided intracranial surgery in resection of a meningioma—a case report. Acta Neurochir (Wien) 164:267–271

48. Kurbegovic S, Juhl K, Sørensen KK, Leth J, Willemoe GL, Christensen A, Adams Y, Jensen AR, Buchwald C von, Skjøth-Rasmussen J, Ploug M, Jensen KJ, and Kjaer A (**2021**) IRDye800CW labeled uPAR-targeting peptide for fluorescence-guided glioblastoma surgery: Preclinical studies in orthotopic xenografts. Theranostics 11:7159–7174

49. Baart VM, Manen L van, Bhairosingh SS, Vuijk FA, Iamele L, Jonge H de, Scotti C, Resnati M, Cordfunke RA, Kuppen PJK, Mazar AP, Burggraaf J, Vahrmeijer AL, and Sier CFM (**2021**) Side-by-Side Comparison of uPAR-Targeting Optical Imaging Antibodies and Antibody Fragments for Fluorescence-Guided Surgery of Solid Tumors. Mol Imaging Biol 25:122–132

50. Boonstra MC, Driel PBAA Van, Keereweer S, Prevoo HAJM, Stammes MA, Baart VM, Löwik CWGM, Mazar AP, Velde CJH van de, Vahrmeijer AL, and Sier CFM (**2017**) Preclinical uPAR-targeted multimodal imaging of locoregional oral cancer. Oral Oncol 66:1–8

51. GEPIA (Gene Expression Profiling Interactive Analysis), http://gepia.cancer-pku.cn/index.html

52. Boonstra MC, Geus SWL De, Prevoo HAJM, Hawinkels LJAC, Velde CJH Van De, Kuppen PJK, Vahrmeijer AL, and Sier CFM (**2016**) Selecting Targets for Tumor Imaging: An Overview of Cancer-Associated Membrane Proteins. Biomark Cancer 8:BIC.S38542

53. Veen M Van, Matas-Rico E, Wetering K van de, Leyton-Puig D, Kedziora KM, Lorenzi V de, Stijf-Bultsma Y, Broek B van den, Jalink K, Sidenius N, Perrakis A, and Moolenaar WH (**2017**) Negative regulation of urokinase receptor activity by a GPI-specific phospholipase C in breast cancer cells. Elife 6:1–20

54. Vincke C, Gutiérrez C, Wernery U, Devoogdt N, Hassanzadeh-Ghassabeh G, and Muyldermans S (**2015**) Generation of Single Domain Antibody Fragments Derived from Camelids and Generation of Manifold Constructs,

55. Muyldermans S (**2021**) A guide to: generation and design of nanobodies. FEBS J 288:2084–2102

56. Vaneycken I, Devoogdt N, Gassen N Van, Vincke C, Xavier C, Wernery U, Muyldermans S, Lahoutte T, and Caveliers V (**2011**) Preclinical screening of anti-HER2 nanobodies for molecular imaging of breast cancer. FASEB J 25:2433–46

57. Arbabi Ghahroudi M, Desmyter A, Wyns L, Hamers R, and Muyldermans S (**1997**) Selection and identification of single domain antibody fragments from camel heavy-chain antibodies. FEBS Lett 414:521–526

58. Romao E, Krasniqi A, Maes L, Vandenbrande C, Sterckx YGJ, Stijlemans B, Vincke C, Devoogdt N, and Muyldermans S (**2020**) Identification of Nanobodies against the Acute. Int J Mol Sci 21:310

59. Schibli R and Schubiger AP (**2002**) Current use and future potential of organometallic radiopharmaceuticals. Eur J Nucl Med 29:1529–1542

60. Dewerchin M, Nuffelen A Van, Wallays G, Bouché A, Moons L, Carmeliet P, Mulligan RC, and Collen D (**1996**) Generation and characterization of urokinase receptor-deficient mice. J Clin Invest 97:870–878

61. Carmeliet P, Schoonjans L, Kieckens L, Ream B, Degen J, Bronson R, Vos R De, Oord JJ Van Den, Collen D, and Mulligan RC (**1994**) Physiological consequences of loss of plasminogen activator gene function in mice. Nature 368:419–424

62. Schneider CA, Rasband WS, and Eliceiri KW (**2012**) NIH Image to ImageJ: 25 years of image analysis. Nat Methods 9:671–675

63. Romao E, Poignavent V, Vincke C, Ritzenthaler C, Muyldermans S, and Monsion B (**2018**) Construction of High-Quality Camel Immune Antibody Libraries,

64. Lemaire M, D’Huyvetter M, Lahoutte T, Valckenborgh E Van, Menu E, Bruyne E De, Kronenberger P, Wernery U, Muyldermans S, Devoogdt N, and Vanderkerken K (**2014**) Imaging and radioimmunotherapy of multiple myeloma with anti-idiotypic Nanobodies. Leukemia 28:444–447

65. Goyvaerts C, Groeve K De, Dingemans J, Lint S Van, Robays L, Heirman C, Reiser J, Zhang XY, Thielemans K, Baetselier P De, Raes G, and Breckpot K (**2012**) Development of the Nanobody display technology to target lentiviral vectors to antigen-presenting cells. Gene Ther 19:1133–1140

66. Gibson DG, Young L, Chuang R, Venter JC, Iii CAH, Smith HO, and America N (**2009**) Enzymatic assembly of DNA molecules up to several hundred kilobases. 6:12–16

67. Breckpot K, Dullaers M, Bonehill A, Meirvenne S Van, Heirman C, Greef C De, Bruggen P van der, and Thielemans K (**2003**) Lentivirally transduced dendritic cells as a tool for cancer immunotherapy. J Gene Med 5:654–667

68. Goyvaerts C, Broos K, Escors D, Heirman C, Raes G, Baetselier P De, Thielemans K, and Breckpot K (**2015**) The transduction pattern of IL-12-encoding lentiviral vectors shapes the immunological outcome. Eur J Immunol 45:3351–3361

69. Loening AM and Gambhir SS (**2003**) AMIDE: a free software tool for multimodality medical image analysis. Mol Imaging 2:131–7

70. Lefranc MP, Pommié C, Ruiz M, Giudicelli V, Foulquier E, Truong L, Thouvenin-Contet V, and Lefranc G (**2003**) IMGT unique numbering for immunoglobulin and T cell receptor variable domains and Ig superfamily V-like domains. Dev Comp Immunol 27:55–77
